# Cyclodextrin nanoparticles for diagnosis and potential cancer therapy: A systematic review

**DOI:** 10.3389/fcell.2022.984311

**Published:** 2022-09-08

**Authors:** Anandakrishnan Karthic, Arpita Roy, Jaya Lakkakula, Saad Alghamdi, Afnan Shakoori, Ahmad O. Babalghith, Talha Bin Emran, Rohit Sharma, Clara Mariana Gonçalves Lima, Bonglee Kim, Moon Nyeo Park, Sher Zaman Safi, Ray Silva de Almeida, Henrique Douglas Melo Coutinho

**Affiliations:** ^1^ Amity Institute of Biotechnology, Amity University Maharashtra, Mumbai-Pune Expressway, Mumbai, India; ^2^ Department of Biotechnology, School of Engineering & Technology, Sharda University, Greater Noida, India; ^3^ Centre for Computational Biology and Translational Research, Amity University Maharashtra, Mumbai-Pune Expressway, Mumbai, India; ^4^ Laboratory Medicine Department, Faculty of Applied Medical Sciences, Umm Al-Qura University, Makkah, Saudi Arabia; ^5^ Medical Genetics Department, College of Medicine, Umm Al-Qura University, Makkah, Saudi Arabia; ^6^ Department of Pharmacy, BGC Trust University Bangladesh, Chittagong, Bangladesh; ^7^ Department of Pharmacy, Faculty of Allied Health Sciences, Daffodil International University, Dhaka, Bangladesh; ^8^ Department of Rasa Shastra and Bhaishajya Kalpana, Faculty of Ayurveda, Institute of Medical Sciences, Banaras Hindu University, Varanasi, India; ^9^ Department of Food Science, Federal University of Lavras, Lavras, Brazil; ^10^ Department of Pathology, College of Korean Medicine, Kyung Hee University, Seoul, South Korea; ^11^ Faculty of Medicine, Bioscience and Nursing, MAHSA University, Jenjarom, Malaysia; ^12^ IRCBM, COMSATS University Islamabad, Lahore, Pakistan; ^13^ Department of Biological Chemistry, Regional University of Cariri –URCA, Crato, Brazil

**Keywords:** targeted delivery, chemotherapy, hydrophobic drug, theranostics, breast carcinoma, biomarker, photothermal therapy, photodynamic therapy

## Abstract

Cancer is still one of the world’s deadliest health concerns. As per latest statistics, lung, breast, liver, prostate, and cervical cancers are reported topmost worldwide. Although chemotherapy is most widely used methodology to treat cancer, poor pharmacokinetic parameters of anticancer drugs render them less effective. Novel nano-drug delivery systems have the caliber to improve the solubility and biocompatibility of various such chemical compounds. In this regard, cyclodextrins (CD), a group of natural nano-oligosaccharide possessing unique physicochemical characteristics has been highly exploited for drug delivery and other pharmaceutical purposes. Their cup-like structure and amphiphilic nature allows better accumulation of drugs, improved solubility, and stability, whereas CDs supramolecular chemical compatibility renders it to be highly receptive to various kinds of functionalization. Therefore combining physical, chemical, and bio-engineering approaches at nanoscale to specifically target the tumor cells can help in maximizing the tumor damage without harming non-malignant cells. Numerous combinations of CD nanocomposites were developed over the years, which employed photodynamic, photothermal therapy, chemotherapy, and hyperthermia methods, particularly targeting cancer cells. In this review, we discuss the vivid roles of cyclodextrin nanocomposites developed for the treatment and theranostics of most important cancers to highlight its clinical significance and potential as a medical tool.

## 1 Introduction

About 10 million cancer related deaths per year are reported worldwide (Fact sheets, 2021). The projected number of new cancers in United States alone is estimated to be 1.8 million. Among them the top three cancers are of breast, prostate, and lung origins (Siegel et al., 2021; Xia et al., 2022), whereas all over the world there was an estimated 19.3 million new cases of cancer in 2020. Almost 9.8 million people died due to the complications related to it. In this review, we aim to focus on the nanotherapy for 5 of top 10 cancers observed worldwide, namely, breast, lung, prostate, liver, and cervical cancers (Sung et al., 2021).

Cancer is a physiologically abnormal state governed by certain hallmarks like self-sufficiency in growth signals, reprogramming of energy metabolism along with evasion of antigrowth signals, apoptosis, and immune response. Most of them are triggered due to inherent or acquired mutations (Hanahan and Weinberg, 2000, 2011). Considering all these points, cancer hallmarks can be defined as “acquired evolutionary-advantageous characteristics that complementarily promote transformation of phenotypically normal cells into malignant ones and promote progression of malignant cells while sacrificing/exploiting host tissue” (Fouad and Aanei, 2017).

For cancer, there are more palliative options than curative treatment. Most of them just manage the quality of life. The current methods like surgery, chemotherapy, and radiotherapy remain challenged by multiple limitations including improper drug targeting and delivery (Awasthi et al., 2018). At the tumor site due to increased angiogenesis there is an unpredictable blood flow and abnormal neovasculature among many other tumor microenvironment factors (like extracellular matrix, pH, and enzymatic concentrations), which impacts drug penetration (Petrova et al., 2018). Chemotherapy is the widely used mode of treatment; however, they cause many side-effects. Moreover, many potent drugs possess poor biocompatibility and solubility (Gidwani and Vyas, 2015). Hence, the efficiency of chemotherapy can be improved by maximizing the amount of anticancer drug reaching the target site. Advancements in drug delivery technology enabled scientists to achieve targeted delivery of anticancer drugs to the tumor site. This is performed by using a vehicle functionalized with different compounds including targeting agents to carry anticancer drug safely without leaking it to other cells (Lakkakula et al., 2021). This gave rise to novel drug delivery systems (NDDS).

Nanotechnology plays a phenomenal role in developing NDDS for cancer therapy (Vij and Wadhwa, 2020). Since nanotechnology deals with the engineering and application of materials within the dimensions of 1–100 nm, smaller and better delivery systems can be fabricated. These nanocarriers could easily enter inside the cancerous cells and cause cytotoxic effects. By transforming the current drug delivery systems into nanoscale, better penetration of drugs could be achieved in manually unreachable histological locations like lung, colon, and brain cancers (Shi et al., 2020). The field of nanotechnology is very exciting because we could exploit the diverse properties of multiple materials, which can be constructed in various shapes and functionalized in tons of ways to attain specificity.

Among such natural materials, cyclodextrins are of special interest in the field of drug delivery and pharmaceutics. Cyclodextrins (CDs) are water soluble carbohydrate oligomers composed of six to eight glucose units in a ring structure. Cyclodextrins have amphiphilic cup-like structures with a hydrophilic exterior and hydrophobic interior. The number of sugar monomers defines the name and dimensions of the toroidal cavity: α-CD, β-CD, and γ-CD, having six, seven, and eight glucose residues, respectively. This differentiates the cyclodextrins by its size, drug carrying capacity, and biological fate. These nano-oligomers have the status of generally recognized as safe (GRAS) given by the United States Food and Drug Administration (US-FDA).

**FIGURE 1:**
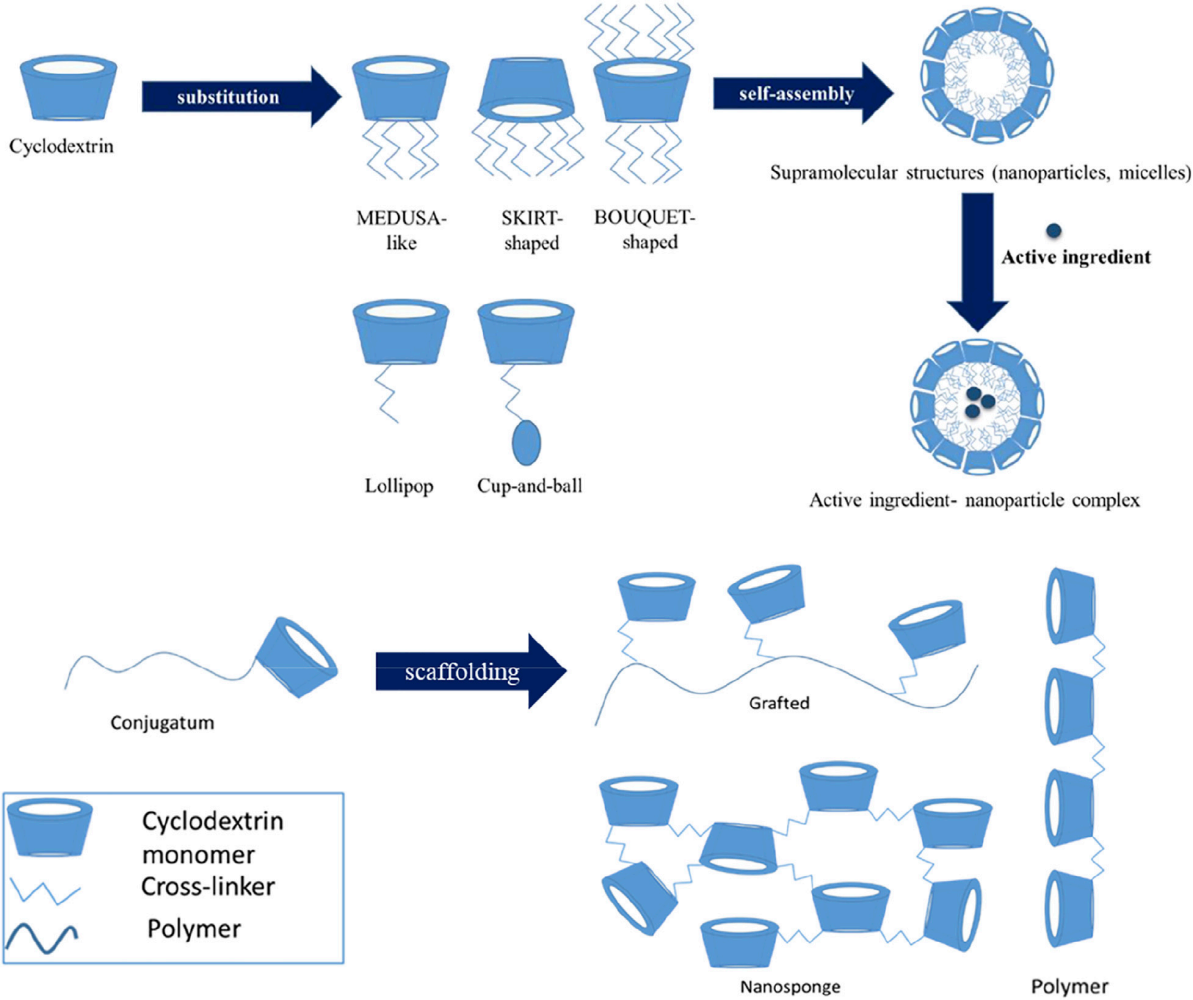
Architectures of CD and CD-based polymers for drug delivery. Haimhoffer et al. (2019).

Many chemical modifications can be carried out to further improve the unique physicochemical properties of CDs. Some of them like hydroxypropyl-β-CD (HP-β-CD) and sulfobutylether-β-CD (SBE-β-CD) among numerous others approved by the US-FDA for pharmaceutical usage possess very high water solubility (Lakkakula and Krause, 2013). Since then, the usage of β-CD in drug delivery and biomedical applications has increased multi-folds. The special properties of CDs which motivates its usage in medicine are high supramolecular chemical compatibility, bioavailability, amphiphilicity, and low toxicity. CDs augment the stability and bioavailability of active pharmaceutical ingredients *via* its host–guest interactions by forming an inclusion complex (IC) (Lakkakula and Maçedo Krause, 2014). Due to this property, various anticancer drugs having hydrophobic nature have been conjugated with CDs as inclusion complexes to improve their bioavailability and systemic circulation. The binding of the drugs is reversible because no covalent bonds are formed during preparation. After administration, the rapid dissociation of drug from inclusion complexes occurs due to dilution by the plasma and extracellular fluids. Thus, the dissociated drug which is separated from its carrier will be rapidly cleared at the rate of free drug from blood circulation, while the CDs will be degraded biologically (Jani et al., 2019).

(Figure 1) shows the exploitation of the cyclodextrin’s amphiphilic properties for designing various kinds of nanostructures for drug delivery. They can be functionalized with multiple charged or neutral polymers to deliver compound of interest to any biological location. One of the most handy properties of CDs is that by adjusting their solubility, hydrophobicity, and targetability *via* supramolecular chemistry drugs can be released in a controlled manner (Yu et al., 2018; Varan et al., 2021). Various types of nanocomposites have been developed and studied such as CD-based liposomes, nanosponges, micelles, nanocapsules, nanoplexes, and nanoparticles for theranostics and therapy (Adeoye and Cabral-Marques, 2017; Ameli and Alizadeh, 2022).

A recent study employing γ-CD and modified lanthanide inclusion complexes having a high biocompatibility was used to detect the anticancer drug irinotecan in liver cancer cells *via* fluorescence changes (Guo et al., 2022). Usage of such smart sensing systems can help us track the selective release of drugs at the tumor with precision. A mannose-modified γ-CD ICs carrying regorafenib could self-assemble into different types of channels to target colorectal cancer cells and elicit cell death. They demonstrated sustained drug release with increased apoptosis along with decreasing tumor supportive factors and pro-inflammatory cytokines (Bai et al., 2021). This study exemplifies that potency of CD in targeting cancer cells can be augmented with usage of suitable targeting agents. Another study involving polycationic β-CD complexed with camptothecin (CPT), a highly potent drug improved the stability of the drug for treatment of both early- and late-stage colon cancers (Ünal et al., 2021). Several other studies show improved permeability, effective tumor site accumulation, and reduced non-specific toxicity (Akkın et al., 2022; Zhang R. et al., 2022).

The main objective of this review is to highlight the role of cyclodextrin-based nanocomplexes to tackle the most frequent (top) five cancers, namely of lung, breast, prostate, cervical, and liver origins. We first brief the *in vitro*, *in vivo* diagnostics, and theranostics studies reported over the years. We then summarize the research employing CD-based nanosystems to deliver different types of cargoes like drugs, chemical agents, and biologics like ribonucleic acid (RNA) and peptides in a tumour targeted manner. Fight against cancer necessitates a multi-pronged approach. Keeping in mind that therapy and patient safety is the main goal, here we also discuss various treatment means used against cancer like chemotherapy, magnetic hyperthermia, gene therapy photothermal, and photodynamic therapy. By reading this review, one could gain a comprehensive introduction to cyclodextrin-based nanodrug delivery systems and the brilliant ideologies used to tackle the world’s most complex disease.

## 2 Cyclodextrins and their advantages

The unique physicochemical properties of cyclodextrins have attracted scientists to use them as a pharmaceutical excipient and drug carrier over the decades. However, the advent of multiple functionalization and nanotechnology has engraved the use of cyclodextrin for hydrophobic drug delivery, especially in cancer therapy (Shelley and Babu, 2018), while the use of CD–drug inclusion complexes by themselves is handy in overcoming several drawbacks of administration of anticancer drugs in free form. Further advantages are found when CD-ICs are combined with additional components in the form of nano-delivery system. These novel CD-based nanosystems impart enhanced antitumor effects when carrying the drug. Exciting results pertaining to higher cellular uptake and higher selectivity to tumor cells are possible when functionalized with suitable agents. Moreover, the masking effects of CDs lead to improved stability, solubility, and bioavailability of drugs. These properties altogether constitute a clear pharmacokinetic profile, supporting the development of new CD-based delivery systems for efficient anticancer therapies (Santos et al., 2021).

**FIGURE 2:**
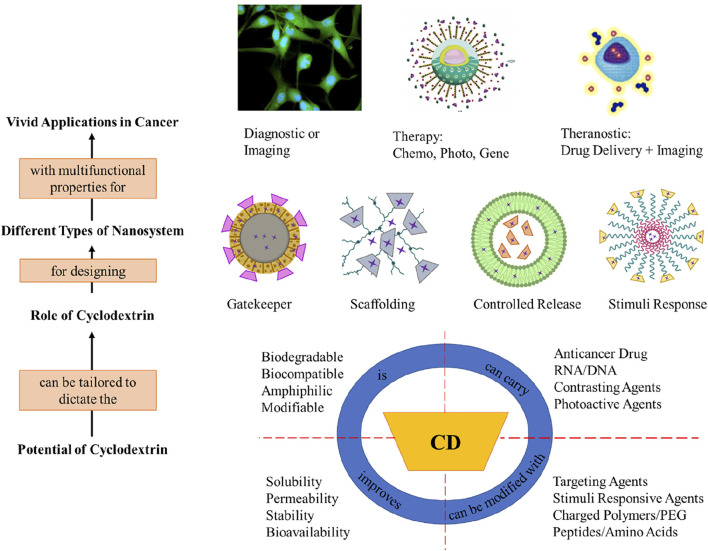
Summary of advantageous properties of cyclodextrin and their applications.

Cyclodextrins are generally not metabolizable by gastric acids or amylase. When CDs are administered orally, it was observed that CDs rapidly hydrolyze in the intestine and get excreted *via* feces (Mu et al., 2022). While in parenteral administration, the primary excretion of CDs occurs *via* urine (Loftsson et al., 2016). However, in some cases, nephrotoxicity and risk of hemolysis are observed (Loftsson and Brewster, 2011). Nevertheless, CD-based delivery systems when associated with other nanotechnological approaches cause negligible harm. Because such strategies of combination derive multifunctional systems, which improve complexation efficiency, increase drug loading capacity, reduce toxicity, extend half-life, provide sustained, controlled, and targeted drug release patterns (Gidwani and Vyas, 2015) (Figure 2) give a graphical summary of resourcefulness of using cyclodextrins in nanosystems for cancer nanotechnology. The properties of CDs which are of particular advantage in designing the nanoplatform for targeted drug delivery and theranostics are emphasized.

## 3 Cyclodextrin nanoparticles for therapy and diagnostic applications

Distinguishing cancerous cells from normal tissues is important for cancer diagnosis. In this regard, nano-engineering can improve the targeting and luminescent properties of various materials enabling them to be used in biomedical applications. This has led to the rise of bioimaging technique to identify different tumors using nanomaterials (Zhang Y. et al., 2022). In this section we discuss variety of cyclodextrin nanocomposites used for various cancer diagnosis and theranostic purposes.

Quantum dots (QDs) have special physicochemical properties, which are widely used in biosensing, bio-imaging, and drug delivery. Most QDs are prepared using semiconductor materials. Zhao et al. (2018) reported a rationally designed CdSe/ZnS QDs with β-CD acting as an external coat to conduct fluorescent imaging in cancerous cells. Doxorubicin (DOX) was encapsulated inside this β-CD-polyamine complex for chemotherapeutic action. The transmission electron microscope (TEM) demonstrated the spherical nature of QDs with 10 nm average size. The intracellular uptake of QDs was studied using confocal laser scanning microscopy (CLSM). It confirmed that NPs internalization occurred in the cells through endocytosis within 3 h and eventually concentrated in the vesicle membranes. This is possibly due to electrostatic interaction between positively charged QDs and negatively charged membranes. Moreover, until 6 h the red fluorescence remained intact after which they became dotted, which indicated the breakage and release of DOX into cytoplasm. Furthermore, the 3-(4,5-dimethylthiazol-2-yl)-2,5-diphenyl tetrazolium bromide (MTT) assay showed increased cytotoxicity in HepG2 cells in addition of polyamine-coated QDs. This is attributed to the polymer’s positive charge, which increased the delivery amount of DOX inside the cells.

A targeted magnetic resonance imaging (MRI) nanocontrast agent was developed by Mortezazadeh et al. (2019) using gadolinium (Gd) NPs enclosed with β-CD-based polyester and folic acid (FA). A nanoparticulate contrasting agent can reach the desired area and improve tissue discrimination for better diagnosis. The polymer coating gave the nanoparticles a longer stability under biological conditions and prevented leakage to normal tissues. The coated spherical GdNPs had diameter in the range of 75–95 nm. MTT assays indicated that these coated NPs unlike free Gd_2_O_3_ were non-toxic toward normal human breast cells (MCF-10A). Their MRI efficiency was tested on both Madison lung (M109) and mouse breast (4T1) cancer cell lines. Since M109 cells overexpress folate receptors, the T_1_ signal in these cells were around four times intense than control, and the T_2_ signal was eight times milder, which improved the contrast in imaging. Whereas 4T1 cells only showed twice the intense T_1_ signal and a 50% less intense T_2_ signal. Furthermore, *in vivo* MR imaging in mice tumors exhibited high contrast-to-noise rates (CNR). From the studies it was concluded that folate-coated NPs were better in tumor localization than the plain NPs due to better targeting along with enhanced permeability and retention effect.

Another similar work used superparamagnetic iron oxide nanoparticles (SPIONs) complexed with bisphosphonate-derivatized-β-CD and curcumin (CUR), which could self-assemble. The system was prepared using the sonication method to where curcumin acted as a cross-linker *via* host–guest interactions with cyclodextrin-modified SPIONs. The resulting monodispersed magnetic nanoparticle complex had a high r_2_ relaxivity (389.96 mM^−1^ s^−1^), which led to excellent T_2_ contrast imaging in mice-bearing 4T1 tumor. The optimum for T_2_-weighted MRI was achieved 4 h post injection of the nanocomplex and stayed the same up to 8 h of time post injection. Alongside, the cytotoxic effect of the curcumin-nanocomplex was significantly higher than free CUR treatment in mice. Moreover, no obvious pathological changes were observed in them (Shen et al., 2020).

On the same line T_1_–T_2_ dual contrast enhancement-based work was carried out by Mansouri et al., by synthesizing magnetic nanoparticles coated with polycyclodextrin (PCD) and Gd^3+^. MNPs were synthesized using the co-precipitation method, whereas β-CD was polymerized in the presence of a cross-linker to form PCD. Other carboxylic and hydroxyl groups were added, which ensured the chelation of Gd ions. Furthermore, CUR was complexed into hydrophobic CD cavities for theranostic application. The hence formed system had a hydrodynamic size of 72 nm, with a zeta potential of around -29 mV. The presence of two types of magnetic metals amidst highly hydrophilic polymers led to increase in the T_1_ signal and significant decrease in the T_2_ signal. The 4T1 tumor in xenografted mice was distinctly detected within 1 h post injection of the MNP/PCD-Gd/CUR nanocomplex. The CUR-containing nanocomplex displayed better tumor reduction in the mice than plain CUR indicating the selective accumulation and EPR effects. No side effects including weight loss was observed (Mansouri et al., 2021).

Gold nanoclusters (AuNCs) are highly luminescent. Wang et al. (2018) enhanced their water solubility by complexing it with β-CD. The size of the β-CD-modified AuNCs measured using the high-resolution transmission electron microscope (HRTEM) was around 3.14 nm. The biocompatibility of the AuNCs toward both normal and cancerous cells was recorded to be as high as 200 μg/ml depicting about 90% viability. According to the luminescent dark field images, AuNCs exhibited red luminescence and was selectively taken up by MGC-803 (gastric cancer) cells even in the presence of GES-1 (normal gastric epithelium) cells thus ascribing the strong permeability of cancer cells. Even after several phosphate-buffered saline (PBS) washes, the nucleuses of cancer cells remained stained, which clearly shows that the NCs could be up taken by the nucleus instead of merely absorbing on the surface of the cells. Hence, such nanocomposites that directly target cancer cell organelles could be used as a probe for real-time cell imaging.

Photochromic compounds such as spiropyrans could be harnessed as a photo-switchable probe due to its tunable fluorescence emission. In one such work, cyclodextrin nanogels were fabricated with spiropyran moiety and 4-amino-7-nitro-1,2,3-benzoxadiazole (NBD), which resulted in spherical nanogels of approximately 200 nm diameter (Deng et al., 2019a). The nanogels when subjected to ultraviolet (UV) light emitted red fluorescence but in visible light due to photoisomerization green fluorescence emerged. However, spiropyran can absorb emission of NBD and undergo Föster resonance energy transfer (FRET), which was efficient even at pH 5–7. Since, cancer cells lie in the extracellular environment of similar pH, this photo-switchable and dual color property of the nanogels was exploited to image Cal27, human tongue cancer cells; 500 μL of nanogel was incubated in Cal27 cells for 4 h and only red fluorescence was observed, indicating the presence of cancer cells. This kind of fast responding nanoparticles can help in rapid diagnosis of tumors.

Many studies suggested the upregulation of alkaline phosphatase (ALP) in the membrane of liver cancer cells. Thus Fan et al. (2021) strategized to utilize ALP-based chemiluminescence (CL) and photodynamic therapy (PDT) for liver cancer theranostics. Mesoporous silica containing a photosensitizer (4-carboxylphenyl porphyrin) capped with β-CD-CL substrate (3-[(2-spiroadamatane)-4-methoxy-4-(3-phosphoryloxy]-phenyl-1,2-dioxetane]dioxetane) inclusion complex was prepared using an ultrasonic ethanol dispersion method. When the nanoparticulate complex was intravenously injected into mice containing SMCC-7721 (human liver cancer cells) tumor, a high CL intensity was observed at the tumor site after 30 min, which lasted until 60 min. This indicates the high selectivity of the nanocarrier, especially CD which protects the leakage of the CL substrate to non-specific areas. The PDT action occurs when the CL substrate reacts with ALP to perform CL resonance energy transfer to the porphyrin photosensitizer, which generates ^1^O_2_ to kill the tumor cells. The mice-bearing tumors receiving this nanoparticular treatment did not lose weight significantly indicating the low side-effects caused.

Circulating tumor cells (CTCs) are sloughed off cancerous cells, which can travel through the body *via* blood vessels. They can be highly metastatic and cause cancer growth in even dissimilar tissues when deposited. Kun et al. (2018) constructed an electrochemiluminescence biosensor containing β-CD-AuNPs, modified graphene and ferrocene-labeled aptamers as a probe for detecting CTCs. This electrochemical biosensor is reusable (up to 6 cycles) and has a rapid detection limit of 40 MEAR (murine liver cancer) cells/mL. Furthermore, biomarker like thrombin was detected at a concentration as low as 5 ng/L. These types of electrochemical sensors can be highly beneficial for early diagnosis of cancer because they have high specificity and selectivity.

A recent study developed platinum-based metallacycle-adamantane complex and capped it with rhodamine-modified β-CD (RBCD) for theranostic application (Ma et al., 2020). The average size of the supramolecular NPs was 22–30 nm as verified by dynamic light scattering (DLS) and scanning electron microscope (SEM) images. Since the emission of the metallacycles overlaps with the absorption of RBCD, efficient FRET takes place. This phenomenon was exploited to measure the progress of nanoparticle delivery and drug release using CLSM. After 1 h of incubation of NPs in HeLa cells, weak red fluorescence alone was observed due to insufficient cell uptake. After 3 h, clear red fluorescence was seen and negligible blue fluorescence which indicates that the fluorescence of metallacycles was quenched by RBCD because the nanocomplex is stable. After 6 h blue fluorescence steadily increased, suggesting the release of the compounds from the nanoparticles to the cells. The intensity of blue fluorescence increased at 8 h signifying the higher rate of release of metallacycles from the NPs. Simultaneously these NPs elicit anticancer activity. The half maximal inhibitory concentration (IC_50_) values of metallacycle-CD nanoparticles were as low as 5.99 µM for HeLa and 7.42 µM for A549 (adenocarcinomic human alveolar basal epithelial) cells. HeLa tumor-bearing nude mice were intratumorally injected 2 mg/kg amphiphilic nanoparticle complex and monitored for 2 weeks. The smallest tumor size and volume was measured in the amphiphilic nanoparticle complex treated mice in comparison with cisplatin-treated mice and other controls. Therefore, these nanoparticles can work as both a clinically capable cytotoxic agent and image contrasting agent when appropriately functionalized.

Another theranostic approach was adopted by Liu et al. (2019) who used mesoporous silica complexed with AuNPs to deliver DOX to the tumors. The 70–80 nm particles seemed to have a flower-pollen like appearance under TEM. Furthermore, β-CD was capped on surface of the nanoparticles for responsive drug release under low pH conditions. In addition to a good cytotoxicity against HepG2 cells the NPs had an ability to show its position through nuclear magnetic resonance (NMR) imaging. This is possible due to enhanced magnetic properties of gold in nanoscale. The signaling intensity was directly proportional to the concentration of the NPs. Therefore, understanding of the phenomenon active in nanoscale can help us in using them as tool for developing new and better drug delivery systems.

AuNP-conjugated β-CD nanocomposite of around 115 nm and −32 mV zeta potential was explored for imaging human glioblastoma (U87) cells. The nanocomposite did not exert cytotoxic or cytostatic effects on U87 and Vero cells up to 100 μg/ml indicating its high biocompatibility. The poly-phenylene-β-CD-poly-ethylene glycol (PPP-g-PEG) containing nanoparticles had successfully targeted U87 cells with a very bright fluorescence in comparison to Vero cells, which demonstrates its selectivity toward cancerous cells. The authors reported dramatic increase in the imaging quality of the tumor cells after the introduction of β-CD complexes, that is, PPP-CD-g-PEG. This is attributed to the improved bioavailability and targeting effects of both the polymers used due to their intrinsic high solubility (Barlas et al., 2019). Furthermore, these nanopolymer composites were subjected to radiosensitivity assay upon U87 cells. Results showed that the polymeric NPs were compatible for eliciting radiotherapy when complexed with nanogold.

Cyclodextrins can carry the desired compound to the biological target and simultaneously improve solubility, stability, and add supramolecular functionalization properties. However, the currently used targets are usually folate receptors or epidermal growth factor receptors. The functionalization of CD nanoparticles should be optimized for other cancer biomarkers and receptors so that tumor can be identified with greater precision (Schick et al., 2021). Furthermore, other than metallic NPs/QDs exploring different kinds of biocompatible fluorescence and luminescent materials can assist as a nanocontrast or bioimaging agents in cancer diagnosis.

## 4 Cyclodextrin nanoparticles for anticancer applications

The role of CDs in pharmaceutical applications has come a long way from being used for masking taste to the current state-of-the-art targeted release (Yuan et al., 2022). The union of CD with nanotechnology has only furthered the capacity of the cyclodextrin-based derivatives. Hence, cyclodextrin has been utilized for multiple purposes in cancer nanotherapy. The most common reason for using CD is their vast capability for functionalization. As the term suggests the conjugation of different group of elements to the nanosystem confer unique and specific properties (Sheng and Kumar, 2022). For example, adding folic acid to the CD–drug inclusion complex will target the (cancer) cells overexpressed with folate receptors. Whereas addition of polymers like PEG gives stability to the nanosystem and assists in controlled release of the drug. Hence, functionalization of the nanosystems opens new avenues to overcome the biological barriers in cancer therapy (Yi et al., 2022). The fascinating property of cyclodextrins is that they can be employed in any role based on structure and configuration of the nanosystem (Figure 2). Simultaneously they aid in improving the drug loading capacity of hydrophobic anticancer agents. In this section, applications of cyclodextrin nanocomposites and nanopolymers for the treatment of the top cancers (breast, lung, liver, cervical, and prostate) have been discussed. Furthermore, we focus on their synthesis, tumor-specific release, therapeutic action, and the possible mechanism behind them. showcases the vivid nanostructures formed by cyclodextrin-based nanoparticles for cancer therapy. (Table 1) contains the bird’s-eye view of the studies reviewed in this work.

**TABLE 1:** Catalog of cyclodextrin nanocomposites and their applications in cancer therapy.

Type of CD	CD nanocomposite	Size of NP	Application	Active ingredient	Cancer cell line	Reference
**β-CD**	CD-CUR NPs	NR	CT	CUR	A549	Zhang et al. (2016)
**α, β, γ-amino-CD**	AuNPs-PEG/CD-CUR	30 nm	CT	CUR	A549	Hoshikawa et al. (2018)
**β-CD**	SPION- β-CD-DOX	185 nm	CT	DOX	A549	Santos et al. (2018)
**β-CD**	Iron oxide-mesoporous silica/DOX-CD/PEG	80 nm	CT	DOX	A549	Lee et al. (2012)
**γ-CD**	γ-CD-metal organic framework-GRGDS peptide/loaded low molecular weight heparin/DOX	150 nm	CT	DOX	A549	He et al. (2021)
**SBE-β-CD**	CD-RES NPs	264 nm	CT	Resveratrol (RES)	A549	Wang et al. (2020)
**SBE-β-CD**	ERL-CD-PLGA NPs and PEI-PLGA-QA	∼198 and ∼205 nm	CT	ERL and quinacrine (QA)	A549	Kulkarni et al. (2021)
**α-CD**	α-CD-PO-CTD	∼196 nm	CT	Costunolide (CTD)	A549	Alhakamy et al. (2022)
**SBE-β-CD**	ERL-CD-PLGA NPs	210 nm	CT	ERL	A549, H517, H460, and H4006	Vaidya et al. (2019)
**β-CD**	QD-β-CD-C-2808	∼150 nm	CT	C-2808	Du-145 and LNCaP	Pilch et al. (2022)
**Sulfated β-CD**	AuNP-TAN/MAN-CD-PEI	100 nm	CT	Tanshinone (TAN) and α-mangostin (MAN)	DU145 and PC3	Qiu et al. (2016)
**β-CD**	Fe_3_O_4_@CD-EDTA@CPT	NR	CT	CPT	HeLa	Rajan et al. (2017)
**β-CD**	Polyethylene glycol@CD polymer@poly (2-(dimethylamino) ethyl methacrylate)	100–450 nm	CT	DOX	HeLa	Zhang et al. (2013)
**β-CD**	β-CD-(PDMAEMA)_7_ at benzimidazole modified poly (ε-caprolactone)	220 nm	CT	DOX	HeLa	Zhou et al. (2018)
**β-CD**	DOX@Mesoporous silica@CD NPs	140 nm	CT	DOX	HeLa	Chen et al. (2014)
**α-CD**	Mesoporous silica@Tm,Yb,Y@CD-DOX	50 nm	CT	DOX	HeLa	Cui et al. (2015)
**β-CD**	MNPs@PAIP-CD-FA	40 nm	CT	DTX	HeLa	Tarasi et al. (2016)
**β-CD**	SmFeO_3_@CD-5FU	52 nm	CT	Fluorouracil (5FU)	HeLa	Hariharan et al. (2019)
**β-CD**	MTX@β-CD@AuNPs	12 nm	CT	MTX	HeLa	Silva et al. (2018)
**β-CD**	Paclitaxel/β-CD@ Poly (Acrylic Acid) NPs	100–200 nm	CT	PTX	HeLa	Yuan et al. (2016)
**β-CD**	pPTX@CD-SPION	221 nm	CT	PTX	HeLa	Jeon et al. (2016)
**HP-β-CD**	FRBE- HP-β-CD ICs	NR	CT	Fermented Egyptian rice bran extract (FRBE)	HeLa	Fahmy et al. (2022)
**HP-β-CD**	FMN-HP-β-CD-PLGA NPs	210 nm	CT	Formononetin (FMN)	HeLa and MCF-7	Guo et al. (2017)
**HP-β-CD**	BAB-HP-β-CD ICs	60 nm	CT	Barbigerone	HepG2	Qiu et al. (2015)
**β-CD**	FA-PEG-β-CD NPs	55 nm	CT	DOX	HepG2	Fan et al. (2019)
**β-CD**	β-CD modified Pt (II) metallacycle-based polymer	300 nm	CT	DOX	HepG2	Chen et al. (2020)
**β-CD**	Fe_3_O_4_@NH_2_-β-CD@GA MNPs	147 nm	CT	Gambogic acid (GA)	HepG2	Fang et al. (2019)
**β-CD**	HAD-β-CD assembly	231 nm	CT	PTX	HepG2	Chen et al. (2016)
**β-CD**	DTX/FA-CD NPs	NR	CT	DTX	HepG2 and HeLa	Xu et al. (2016)
**β-CD**	DTX-CD-calixarene	35 nm, 150 nm	CT	DTX	LNCaP	Gallego-Yerga et al. (2017)
**methylated-β-CD**	DTX-CD-calixarene nanospheres/nanocapsules	100 nm	CT	DTX	LNCaP, PC3	Gallego-Yerga et al. (2021)
**β-CD**	β-CD-grafted poly (ethylene glycol)/poly (l-glutamic acid)	80 nm	CT	CPT	MCF-7	Du et al. (2014)
**γ-CD**	CUR-γ-CD liposomal nanoparticles	67 nm	CT	CUR	MCF-7	Dhule et al. (2012)
**β-CD**	β-CD-CUR/Maltogenic amylase	NR	CT	CUR	MCF-7	Roozbehi et al. (2021)
**α-CD**	α-CD-based polyrotaxanes-poly (DOX)-co-poly [(ethylene glycol) methyl ether methacrylate]	63 nm	CT	DOX	MCF-7	Bai et al. (2018)
**β-CD**	PEG/β-CD/methylacrylate unimolecular micelles	56 nm	CT	DOX, CPT	MCF-7	Gao et al. (2019)
**HP-β-CD**	DTX @modified polycaprolactone@HP-β-CD	74 nm	CT	DTX	MCF-7	Mu et al. (2022)
**HP-β-CD**	Niosome-HP-β-CD-6G	180 nm	CT	6-gingerol (6G)	MCF-7	Mazyed et al. (2022)
**γ-CD**	γ-CD@ phenylacetic acid at2,3-dimethylmaleic anhydride@poly (ethylene glycol)@transferrin	120–134 nm	CT	Topotecan	MDA-MB-231	Yoon et al. (2020)
**β-CD**	Au-GS@β-CD nanoclusters	3.14 nm	CT	NA	MGC-803	Wang et al. (2018)
**SBE-β-CD**	Liposome/SBE-β-CD- β-lapachone	190 nm	CT	β-lapachone	PC3	Wu et al. (2020)
**β-CD**	β-CD-CDI-FLT	∼99 nm	CT	Flutamide (FLT)	PC3	Allahyari et al. (2021)
**β-CD**	CPT-β-CD nanosponges	400 nm	CT	CPT	PC3, DU145	Gigliotti et al. (2016)
**β-CD**	CUR-CD-cellulose nanocrystals	206 nm	CT	CUR	PC3, DU145, HT29	Ntoutoume et al. (2016)
**β-CD**	PLGA-β-CD-MTX	70–200 nm	CT	MTX	T47D	Gorjikhah et al. (2017)
**β-CD**	Acetylated-β-CD-poly (2-ethyl-2-oxazoline)-Fe_3_O_4_	20 nm	CT/hyperthermia	DOX	MCF-7	Soleimani et al. (2021)
**β-CD**	Amphiphilic cyclodextrin@ZnPc/DTX	200 nm	CT/PDT	DTX and zinc phthalocyanine (ZnPc)	HeLa	Conte et al. (2016)
**β-CD**	CuS@CD@DOX/adamantine-RGD	18 nm	CT/PTT	DOX	HeLa	Lu et al. (2018)
**β-CD**	Fe_3_O_4_@PDA@SH-β-CD	8–14 nm	CT/PTT	DOX and polydopamine	HepG2	Mrówczyński et al. (2018)
**Acetalated-β-CD**	Pth-PEG-CD-DOY	229 nm	CT/PTT	Doxycycline	HepG2	Zheng et al. (2021)
**β-CD**	MGO-Fe_3_O_4_-β-CD-cholic acid-hyaluronic acid	15 nm	CT/PTT	CPT/MGO	BEL-7402	Wen et al. (2021)
**β-CD**	Spiropyran-modified β-cyclodextrin nanogel	200 nm	Diagnostic: cell imaging	NA	Cal27	Deng et al. (2019b)
**β-CD**	(β-CD-AuNPs)/graphene	100 nm	Diagnostic: electrochemiluminescence	NA	MEAR, HeLa, and HL-60	Kun et al. (2018)
**β-CD**	GdNPs@β-CD@folic acid	100 nm	Diagnostic: MRI nanocontrasting	NA	M109 and 4T1	Mortezazadeh et al. (2019)
**β-CD**	UCNP/COOH-β-CD/Ad-ZnPc	85 nm	PDT	Adamantine phthalocyanine	HeLa	Wang et al. (2016)
**β-CD**	FA-PEG-β-Ce6	<300 nm	PDT	Chlorin e6 (Ce6)	HeLa	Kim et al. (2021)
**β-CD**	Pheophorbide-adamantanyl-FA-CD	200–300 nm	PDT	Pheophorbide	MCF-7, PC3	Zagami et al. (2019)
**β-CD**	CD-PLL/PEG-TPP/DNA	90 nm	Photodynamic gene therapy	rev-casp-3	HeLa	Gao et al. (2017)
**α-CD**	Fe_3_O_4_@Cu_2-x_S-α-CD-Ce6	∼5 nm	PTT/PDT	Ce6	HepG2	Zhang et al. (2021)
**SC**_**12**_ **CD clickpropylamine**	siRNA-CD-GALA peptide-PEG	200 nm	RNA interference	ZEB1 and NRP1 siRNA	PC3Luc and PC3	Evans et al. (2017)
**β-CD**	SPION-bisphosphonate-derivatized-β-CD-CUR	180–300 nm	Theranostic	CUR	4T1	Shen et al. (2020)
**β-CD**	MNP/PCD-Gd/CUR	57 nm	Theranostic	CUR	4T1	Mansouri et al. (2021)
**β-CD**	Metallacycle-Rhodamine modified β-CD	22–30 nm	Theranostic	Metallacycle-adamantane	HeLa	Ma et al. (2020)
**β-CD**	CdSe/ZnS/β-CD quantum dots	10 nm	Theranostic	DOX	HepG2	Zhao et al. (2018)
**β-CD**	DOX@β-CD-AuNPs	70–80 nm	Theranostic	DOX	HepG2	Liu et al. (2019)
**β-CD**	AuNPs@poly (p-phenylene-β-cyclodextrin)-graft-poly (ethylene glycol)	115 nm	Theranostic	NA	U87	Barlas et al. (2019)
**β-CD**	Mesoporous silica-(4-carboxylphenyl porphyrin) capped with β-CD-CL substrate	70 nm	Theranostic	4-carboxylphenyl porphyrin	SMCC-7721	Fan et al. (2021)

NR: not reported, NA: not applicable, CT: chemotherapy, PDT: photodynamic therapy, PTT: photothermal therapy, DOX: doxorubicin, PTX: paclitaxel, DTX: docetaxel, ERL: erlotinib, CPT: camptothecin, CUR: curcumin. for other abbreviations and cell line name please refer to the main text.

### 4.1 Breast cancer

There are numerous lifestyle and biological factors like gene mutations, hormonal changes, familial history, age, reproductive behavior, diet, physical activity, and exposure to mutagens, which might lead to breast tumors (Coughlin, 2019). All of them are treatable, whereas some may even be prevented by adopting healthy practices. It is now possible to use nanotechnology-based treatments to kill breast tumors. Recently an attempt to treat triple-negative breast cancer (highest mortality rate subtype) was studied using nanoparticle-based delivery of siRNA. It successfully reduced the tumor growth rate by causing POLR2A gene suppression (Xu et al., 2019).

Camptothecin (CPT) is a pentacyclic alkaloid exhibiting anticancer activities against lung, prostate, breast, colon, and melanoma cancers. Nonetheless, it has poor aqueous solubility and severe side effects. It is confirmed that the lactone ring form of the drug is active, and the carboxylate form is toxic to the normal cells. Above pH 7, it is observed that the equilibrium of CPT shifts toward non-active form of the drug. So, to protect the hydrolysis of the drug, Du et al. (2014) created a polymer-based drug carrier system; β-CD-grafted poly (ethylene glycol)/poly (l-glutamic acid) or mPEG-PLG-CPT@CD. These nanoparticles were observed to be spherical, with an average size about 80 nm under TEM. Zeta potential was measured to be -9.14 mV, which suggested their prolonged circulation in blood as opposed to the positively charged particles, which are liable to precipitation in serum. To check the protective effect of the NPs on the drug, reversed phase analytical high performance liquid chromatography (RP-HPLC) was performed in 7.4 pH physiological buffer at 37°C. Results confirmed that free CPT hydrolyzed rapidly and only 20% of it maintained the lactone ring structure after 12 h incubation, whereas the nanoparticulate CPT was nearly unreduced. Cumulative release studies showed linear gradual profiles in 150 h, in which only 24.5% of CPT was released until 24 h due to the sustained drug release behavior of the diblock polymer NPs. Chemical stimulated release behavior was analyzed when adamantane carboxylate (ADC) was added to the PBS, which showed a slightly accelerated process of drug release specially after 30 h. MTT assay showed dose-dependent cytotoxicity of CPT containing NPs. However, it had lower cytotoxicity than free CPT, which was maybe due to gradual drug release from the polymeric NPs. However, the application of controlled release is encouraged for enhanced long-term activity.

A group used PLGA-β-CD to encapsulate methotrexate (MTX), an antifolate that competitively inhibits dihydro folate receptor (DHFR) which is necessary to synthesize purines and pyrimidines thereby stopping DNA/RNA synthesis in cancer cells leading to its death. Poly (lactic-co-glycolic acid) (PLGA) is a known biocompatible and biodegradable polymer having controlled drug release properties. The PLGA-β-CD copolymer nanoparticles after the loading of drug were observed to be spherical under SEM. Size of the NPs ranged from 70 to 200 nm. A high encapsulation efficiency (EE) of 80% was achieved with a loading capacity (LC) of 18% due to application of CD. T47D breast cancer cell line was tested against plain MTX and loaded PLGA-β-CD in the time periods of 24, 48, and 72 h. The nanoparticulate system showed better cytotoxicity due to controlled release effect. IC_50_ readings were 0.318, 0.294, and 0.241 mg/ml for the copolymer encapsulated MTX vs. 0.391, 0.361, and 0.285 mg/ml for free MTX at 24, 48, and 72 h, respectively (Gorjikhah et al., 2017).

Formononetin (FMN) is known to inhibit tumor cell migration, propagation and invasion, and induce apoptosis in breast, prostate, and cervical cancer cell lines. Weak water solubility and rapid glucuronidation and sulfation, limits the drug’s clinical application. So, this study on FMN containing HP-β-CD ICs was encapsulated into PLGA NPs to check its efficacy as a drug delivery system. The NPs were spherically sized about 210.23 ± 3.4 nm, and the EE was calculated to be 91.97 ± 0.79%. The cumulative release studies showed that drugs in NPs had a sustained release effect resulting in only 50% in 24 h. Cytotoxicity assay, WST-1 colorimetric method using the nanocarrier-drug showed reduction in viability of HeLa and MCF-7 cells above the concentration of 25 µM. The desired bioactivity was not seen due to the incomplete release of FMN from the nanocomplex (Guo et al., 2017). Hence, more appropriate smart polymers exerting timely release of payload should be developed and studied.

Docetaxel (DTX) is an anticancer drug inhibiting microtubule polymerization, but it is insoluble in water. Methyl ether PEG (mePEG)-modified polycaprolactone (PCL) coated with HP-β-CD was used to exhibit controlled release of the drug. PCL protects it from early degradation and mePEG gives the scaffold. The composition led to formation of nanospheres sized 74 nm for mePEG-PCL-CD in diameter. Increased amphiphilic property of mePEG-PCL copolymer is attributed to the smaller size of nanospheres. The complete *in vitro* drug release for mePEG-PCL-CD took 12 h. Also, the nanospheres anticancer efficacy was roughly equivalent to plain DTX solution against MCF-7 cancer cells. Therefore, these nano-sized cyclodextrin carriers of drugs can perform controlled drug release of hydrophobic drugs to the cancer cells and the usage of plain drug solutions, which causes several side effects can be eliminated (Varan and Bilensoy, 2014).

6-gingerol (6G) is a prevalently found secondary metabolite in ginger. It is suggested to possess anticancer activities along with antioxidant and anti-inflammatory effects. Hence, Mazyed et al. (2022) attempted to develop a flexible niosome functionalized with HP-β-CD carrying 6G molecules employing the ethanol injection method (Mazyed et al., 2022). These ‘ethoniosomes’ sized around 180 nm had an excellent maximum EE and LC, that is, around 90 and 95%, respectively. The increased permeability and stability of the CD-modified ethoniosomes carrying 6G had IC_50_ of 20.10 ± 0.51 µM as tested upon MCF-7 cells *via* MTT assay. Innovative attempts like this to repurpose naturally available secondary metabolites will add to the options available for treating cancer.

Gao et al. (2019) fabricated unimolecular micelles using PEG, β-CD, and methylacrylate polymers to obtain DOX and CPT prodrug assembly for controlled release purpose. Under TEM observation, the micelles showed a spherical shape with a diameter of 56 nm. Then, the drug release profile at pH 7.4 and 5 containing glutathione (GSH) induced 75% CPT release proving the reduction sensitivity of the micelles. The DOX release was approximately 70% after 72 h at pH 5 but less than 20% at pH 7.4, suggesting the micelle’s pH-controlled release property. Reduction of disulfide linkages is attributed to the CPT release, whereas protonation of docosapentaenoic acid (DPA) chain for the DOX release both of which are possible in the tumor microenvironment. Anticancer efficacy was assessed by MTT assay on MCF-7 cells whose viability reduced to 18% after 48 h incubation with DOX–CPT micelles. IC_50_ value of 2.1 μg/ml was achieved due to the dual drug synergistic effect. In addition, 3D tumor spheroids study of MCF-7 under different pH and GSH levels resulted in a strongest fluorescent signal at pH 5, and 10 mM GSH levels indicates the dual responsive nature of micellar NPs. Multi-responsive nanoparticles can aid researchers in tackling cancer in a better way by improving the targeting and release of drugs.

Another innovative method is the use of CDs as polyrotaxanes reported by Bai et al. (2018). They used hydrazone bonding to attach DOX to ethyl glycinate methacrylamide (MGMA) such that it formed a prodrug, which is pH sensitive (PRMO). Hence, polyrotaxanes with pH-responsive side chains containing DOX was established, which formed micelles of approximately 63 nm whereas in TEM it was 41.1 nm. The synthesis scheme of the nanoparticulate polyrotaxanes containing DOX Enhanced permeation and retention (EPR) effect further enhances the pH-responsive release of the drug contents specifically at the tumor site in the animal model. Cumulative release studies were performed (for 70 h) to test the efficiency of prodrug to active drug conversion, which resulted in less than 10% at pH 7.4 and 6.8 whereas around 70% at pH 5. The activity of the formed nanostructure (10 μg/ml) was tested using MTT assay leading to only 3% viability of MCF-7 cells after 72 h of incubation. These micelles were intravenously subjected to healthy rats and high blood compatibility was observed. Further *in vivo* trials on the rats bearing MCF-7 tumor were tested with control, free DOX, and micellar NPs for 2 weeks. Both free DOX and DOX NPs reduced tumor size around 65% but free DOX also led to body weight loss of mice due to non-specific toxicity. Also, hematoxylin and eosin (H&E) staining in liver, spleen, lung, and kidney of mice indicated that free DOX caused cellular damage unlike the nano-DOX. Capability of a drug for killing tumors and exhibiting lower side effects is of great importance. Development of nanomicelles like these is a step taken toward this direction.

Curcumin (CUR) is known to exhibit anti-invasive, anti-angiogenic, and antiproliferative activity against many types of cancer. However, its systemic availability is poor due to its hydrophobicity. Liposomal nanoparticles are known for its ability to retain hydrophobic drugs in their bilayer region of the vesicles; however, only a small amount of drug could be delivered. Hence, to increase the drug loading capacity, CDs were used to contain the weakly hydrophilic curcumin inside them, so that CD–drug complex could be encapsulated inside the liposomal cavity. After formulation of this drug–CD–liposomes, their size was measured to be 67 nm by cryo-TEM. EE was found to be 50%, whereas the conventional curcumin liposomes recorded only 30%. IC_50_ of CD-based liposomes was found to be 11.5 ± 1.1 for the MCF-7 (human breast cancer cell line). Hoechst staining indicated the formation of apoptotic bodies and chromatin condensation. Detection of cleaved poly-ADP ribose polymerase (PARP) further confirmed apoptosis (Dhule et al., 2012).

Roozbehi et al. (2021) designed a curcumin/cyclodextrin system wherein an enzyme like maltogenic amylase (MA) was used to improve the drug release from the CUR/CD inclusion complexes. After achieving the maximum EE and LC of 34.12 and 2.84%, respectively they conducted *in vitro* drug release studies. In absence of MA the drug release was slow and after an initial burst some amount was unreleased, but in the presence of different concentrations of MA the CUR release was rapid and complete. This enhanced release is attributed to the ability of MA to cleave internal α-D-(1,6) and α-D-(1,4) glycosidic bonds. Fluorescent microscopy indicated an increased uptake of CUR into MCF-7 cells in presence of MA when compared to untreated CUR/CD. MTT cytotoxicity assay was conducted with free CUR, CD/CUR, and CUR/CD/MA, only the latter two could significantly inhibit the cancer cell growth with an IC_50_ of 60 and 1 µM, respectively. Furthermore, acridine orange/ethidium bromide (AO/EB) staining showed that the enzymatically released CUR induces apoptosis and necrosis in MCF-7 cells thereby killing the cancer. Using such enzyme-based drug delivery systems could help in the complete release of anticancer drug at the tumor site.

A multifunctional nanoparticle based on γ-CD was developed by conjugating it with PEG, phenylacetic acid (PA), 2,3-dimethylmaleic anhydride (DMA) and transferrin (Tf) to deliver an anticancer drug topotecan. The size of these spherical nanoparticles ranged from 120 to 134 nm as visualized in FE-SEM (in both 7.4 and 6.0 pH). However, there was an increase in zeta potential from −26.7 to −11.6 mV most probably due to cleavage of DMA. The *in vitro* cumulative drug release studies were conducted at pH 7.4 and 6.0. Almost two times faster and higher drug release rates were seen at lower pH due to the pH-responsive nature of DMA. Fluorescence microscopy showed that the NPs accumulated in Tf positive MDA-MB-231 cells but comparatively very less in CHO-K1 (Tf negative) cells. This indicated the role of transferrin in tumor cell specific targeting. Similarly, high cytotoxic activity was exhibited by the nanocomplex against MDA-MB-231, whereas 80% of CHO-K1 cells were still viable; 2.5 mg/kg of nanoparticles were intravenously injected to MDA-MB-231 tumor-bearing nude mice and monitored for 7 days. The tumor volume was 6.7-fold smaller in the treated mice as compared to control. These results suggest that targeted delivery of NPs can preferentially bind *in vivo* tumor cells and enhance the activity of anticancer drugs (Yoon et al., 2020).

Soleimani et al. (2021) synthesized magnetic nanohydrogels capable of responding to both pH and GSH to treat breast cancer *via* chemo/hyperthermia. The highly functionalized nanogels were prepared using multiple steps including co-precipitation, distillation, and sol–gel method. β-CD was first iodinated then acetylated and crosslinked with poly (2-ethyl-2-oxazoline) to get conjugated with Fe_3_O_4_ NPs. The magnetic hydrogels possessed a good EE and LC values upon loading of DOX drug. Under the conditions of GSH 10 mM and pH 5.3 the drug release from nanogels were 89.2%, which was attributed to the dual-responsive nature of the nanosystem due to breakage of crosslinking and swelling of gels, respectively. The activity of controlled release of the drug from the nanogel along with hyperthermia was assayed on MCF-7 cells using the MTT method over a period of 48 h. Due to burst release and free roaming, plain DOX had a lower IC_50_ values at all concentrations compared to nanogels at 24 h time point; however, at the end of the assay the nanogels at concentration 4 μg/ml and above elicited higher cell death. Although the nanogels had stimuli-responsive drug release, faster unloading could help in treating the tumor area better.

While treatment options for breast cancers are increasing, the more aggressive triple-negative breast cancer (lacking estrogen, progesterone, and human epidermal growth factor receptors) needs further research to find novel receptors and biomarkers for targeting (Jain et al., 2020). Novel drug combinations and multi-responsive nanoparticles functionalized with cyclodextrin for controlled release could help in resolving this problem.

### 4.2 Cervical cancer

Among gynecological cancers, cervical cancer is the most common cause of death in women, especially from developing regions. A strong causative agent of this cancer is human papilloma virus (HPV), which is transmitted through sexual intercourse. Spreading awareness could prevent this type of cancers; however, lakhs of women have been diagnosed since 2018, for whom nano-therapy could help fight the disease (Momenimovahed and Salehiniya, 2017).

Paclitaxel (PTX) is one of the most common broad-spectrum anticancer drugs; however, its therapeutic outcome in clinics is reduced due to its low water solubility. Poly(acrylic acid) or PAA a double-hydrophilic polymer and β-CD were used to construct a comb like polymer, which could carry PTX as multivalent inclusion complex. Due to partly encapsulation of PTX in CD it was hypothesized that polymeric NPs could have a hydrophobic PTX core and water loving PAA surface. NPs were near spherical as shown by TEM with a size range of 100–200 nm. Optimized condition for highest solubility of PTX was achieved at 7.5:1 (β-CD:PTX) feeding ratio containing 36 µg of drug. The *in vitro* release of the NPs for 120 h (at pH 7.4) resulted in fast release up to 60% during first 12 h and thereafter a sustained steady state release. The pharmacological activity of the released drug was evaluated by MTT assay on HeLa cells in comparison to free PTX. Both of them showed growth inhibitory activity killing around 90% cells at 1 mg/ml. *In vivo* biodistribution of the NPs were measured using NIR imaging in mice, which clearly indicated signals in the liver at 3 h; however, with time the signal decreased and went to belly and intestine suggesting its biliary excretion. Meanwhile the fluorescence was retained up to 144 h in tumor to the EPR effect. These results encouraged *in vivo* studies using H22 (murine hepatic carcinoma cells) transplanted mice; 10 mg/kg of plain PTX and NPs were injected to separate mice. The slowest tumor growth and smallest tumor volume were exhibited by NPs treated mice until 17 days. Also the survival rates were best for NP-treated mice with 50 days’ life as compared to 41 days for plain PTX (Yuan et al., 2016).

Fahmy et al. (2022) explored the properties of fermented Egyptian rice bran extract (FRBE) for their anticancer and antioxidative characteristics. Solubility and bioavailability of the phenolic chemical compounds found in the extract was improved after it was complexed with HP-β-CD using the thin-film hydration method. Owing to the FRBE’s increased stability in CD-ICs its *in vitro* antioxidant and anticancer activity was radically higher than plain FRBE. The IC_50_ of the FRBE-CD-ICs was 0.5 ± 0.01 μg/mL as measured by SRB assay against HeLa cells. In addition, its Trolox equivalent antioxidant capacity (TEAC), a standard to measure the antioxidant capacity was as high as 151.30 μM TE/mg complex. Therefore, it is encouraging to explore the medicinal properties of natural extracts for discovering anticancer agents, which can be effectively delivered using nanocarriers like modified cyclodextrins.

pH responsivity is a very suitable strategy to deliver anticancer drug due to tumor microenvironment properties. 2-(dimethylamino) ethyl methacrylate (DMAEMA) is one such polymer that gets protonated under acidic conditions. This was exploited by Zhang et al. (2013) to devise a star like polymer along with PEG and methylacrylate substituted β-CD. Two ratios of poly (D,L-lactide), that is, PLA and star polymer were studied whose size ranged from 100 to 450 nm. DOX was used to evaluate the drug-loading capacity of NPs with 3:10 feed ratio (PLA: star polymer), which equaled to 18% with an EE of 61%. The size observed using TEM in phosphate-buffered saline, PBS (pH 7.4) and acetate-buffered saline (ABS, pH 5.0) was recorded to be 10–20 nm and 20–40 nm, respectively without DOX, whereas after loading it, diameter increased to 40 and 100 nm, respectively. The drug release studies were also conducted to assess its pH-based release, and as expected the cumulative release was faster and higher in ABS than PBS due to electrostatic repulsion of protonated DMAEMA chains and higher solubility of DOX in lower pH. The uptake of the released drug was observed under flow cytometry and CLSM; 2 h incubation of nanoparticles in HeLa cells showed enhanced fluorescence. Free DOX was mainly localized inside nucleus due to its passive diffusion and high affinity toward DNA. However, DOX from NPs was present in both nucleus and cytoplasm due to the non-specific endocytosis pathway and endosomal pH response. Cytotoxicity evaluation performed on HeLa cells by CCK-8 assay gave the IC_50_ value of 0.62 μg/ml for the nanocomplex. Therefore, this star carrier can be used as an intracellular pH-triggered anticancer drug release vehicle.

Another example of pH sensitive micelle is based on modified β-CD and poly(2-(dimethylamino)ethyl methacrylate), which hosted DOX as the model drug. Spherical morphology of the nanoconjugate was confirmed by TEM, and the average hydrodynamic size recorded was 220 nm. The drug LC and EE reached up to 40 and 85% in this preparation due to large capacity of β-CD and hydrophobic micelle PCL core. Also the zeta potential was around +5 mV at pH 7. The drug release at pH 5.2 was around 90% due to protonation of amino and benzimidazole groups, which caused dissociation of the complexes. Cytotoxic effect of micelles was tested on HeLa cells using MTT assay at 1 mg/ml concentration (containing 200 µg of DOX), which killed 60% of cells. Whereas free DOX of same concentration killed only 50%, which shows superiority of the drug loaded micelles (Zhou et al., 2018). Polymers like these possessing pH responsive activity can be well exploited for drug delivery at different tumor locations.

PDT effects are limited due to its inability to penetrate large or deeply located tumors. Hence, phototherapy in combination with other therapeutic agents is desirable. In this study Gao et al., used poly(l-Lysine) modified β-CD and PEGylated tetraphenylporphyrin (TPP) platform to carry a gene (rev-casp-3) by electrostatic interactions (Gao et al., 2017). The formed nanosystem had an average spherical diameter of 90 nm as confirmed by TEM and DLS. However, their size increases to nearly 450 nm after DNA complexation. Photodynamic activity of the nanosystem was demonstrated by 9,10-anthracenediyl-bis(methylene) dimalonic acid (AMDA), which traps singlet oxygen (^1^O_2_) produced by TPP. Moreover, the gene transfection efficiency was evaluated using luciferase assay, which suggested the ratio of 10:10:1 (β-CD-PLL/PEG-TPP/DNA) as optimal. The entry of DNA-NP complex into HeLa cells was improved due to electrostatic interaction between positively charged NPs and negatively charged cell membrane. Standard MTT assays was conducted with and without irradiation (40 MW/cm^2^, 400–700 nm, 20 min), obtaining cell viabilities below 40% in 17.5 μg/ml concentration of nanocomplex. The synergistic action of gene-photodynamic therapy causing apoptosis in more than 26.57% HeLa cells was observed. The employment of such combined therapies can improve the effectiveness of the nanocomplexes used in cancer treatment.

A multifunctional nanocomplex for FR-targeted delivery of photosensitizer with ROS sensitivity was developed by Kim et al. (2021) for eliciting PDT upon HeLa cells. For the purpose FA-PEG was conjugated with β-CD, which was further linked using diselenide to the photosensitizer chlorin e6 (Ce6) *via* dialysis method. The average size of the formed nanocomplexes was less than 300 nm. Under dark conditions the nanocomplex containing Ce6 did not show any significant toxicity up to 5 μg/ml concentration. *In vivo* tumoxenograft study of mice-containing HeLa cells upon administration of the nanophotosensitizer and free Ce6 was conducted. The former accumulated specifically at the tumor site, indicating the targeted behavior of the nanosystem. Moreover, the highest tumor inhibition rate upon irradiation of light was seen in the mice injected with multifunctional nanophotosensitizer partially due to ROS generation too.

Tarasi et al. (2016) studied poly-N-5-acrylamidoisophthalicacid grafted onto Fe_3_O_4_ functionalized with β-CD, folic acid, and an amide polymer for delivering docetaxel. The size of the NPs on average was 40 nm. Their EE and LC were 75 and 15%, respectively. Cumulative drug release at pH 7.4, 37°C for 100 h was conducted, in which about 40% release was achieved within 24 h, and thereafter the release was slow and reached a maximum of 71%. Fluorescence microscopy confirmed the folate receptor mediated uptake of the magnetic nanoparticles (MNPs). HeLa cells were used to conduct MTT assay. DTX-loaded NPs showed better cytotoxicity at 24, 48, and 72 h due to better targeting activity in presence of folic acid.

SPIONs were used along with β-CD and polymerized paclitaxel for creating a new delivery system (Jeon et al., 2016). Zeta potential of −28.1 mV was observed in pPTX/CD-SPION, which is suitable for intravenous delivery as they formed a nanoassembly sized around 221 nm. MTT assay was performed on HeLa cells with and without magnetic field, where pPTX/CD-SPION showed better cytotoxic activity in the presence of magnetic field in comparison to free PTX. Then, the free drug and nano-drug was injected to mice containing tumors with exposure to external magnetic field. Largest tumor suppression was seen in pPTX/CD-SPION due to the magnetic guiding and enhanced accumulation. In this respect, magnetic nanocomplexes have a great potency in targeted therapy of tumors.

Mesoporous silica NPs (MSNs) were fabricated along with pH sensitive groups, targeting molecules, and fluorescein labeling. DOX was constituted as the core in the multifunctional NPs whose loading capacity was 12.26%, and β-CDs were used as gatekeeper molecules outside the silica core. The resultant nanoparticles sized 140 nm on average. Results reflecting *in vitro* release behaviors of the DOX-loaded MSN was investigated at pH values of 5.3, 6.8, and 7.4 corresponding to physiological conditions of late endosome, tumor extracellular environment, and blood, respectively, which were 80, 30, and 15%. The reason for this selective release is attributed to the pH sensitive benzoic-imine bond of the β-CD molecules. The DOX-loaded MSN with FA exhibited the lowest IC_50_ of 1.08 mg/ml in HeLa cells as compared to MSN without FA (4.56 mg/ml). The flow cytometry and confocal laser scanning microscopy studies showed that these MSNs have high fluorescence intensity and could track the position of the MSNs owing to the presence of fluorescein (Chen et al., 2014). Hence, such nanocomplexes can be used to both target and trace the anticancer drug at the tumor site.

Another group grafted amphiphilic molecules by using C_16_ alkylthio tails and ethylene glycol groups on the inner and outer side of CD. This led to a vesicle-shaped assembly of the CDs, which were designed to contain DTX and ZnPc (photosensitizer). The hydrodynamic size was approximately 200 nm and drug LC was 5%. The stability of the prepared NPs was observed to be high as it showed negligible aggregation after 72 h of incubation in PBS. The witnessed hemolytic activity of the NPs was less than 10%. Combined chemo- and photo-therapy was tested using HeLa cells at 10 μg/ml. The cell viability was reduced by 30% due to the photodynamic effect, and at 72 h nuclear fragmentation was observed indicating DTX-induced cell death (Conte et al., 2016). Thus, the usage of combination treatment in nanoform is encouraged for better treatment efficiency.

Silva et al. (2018) obtained a ternary nanosystem formed from MTX, β-CD inclusion complex capped on AuNPs. TEM micrographs depicted the spherical morphology of both plain AuNPs and that coated with β-CD/MTX. Dynamic light scattering (DLS) measurements were 19 nm for plain AuNPs and 17 nm for IC capped AuNPs. The hydration sphere measured by DLS appeared as citrate and associated ions, whereas decreased hydration sphere in IC-conjugated AuNPs was corresponded to the high capacity of CD to bind water molecules *via* hydrogen bonding. Then, the cell viability of HeLa cells was analyzed comparing free MTX and IC by MTT assay (for 72 h), obtaining IC_50_ values of 0.0839 and 0.239 mM, respectively. This increased IC_50_ value indicated the protective effect of CD due to inclusion of pteridine ring of MTX inside the cavity. At last, the IC-NPs were tested on HeLa cells with 15 min irradiation of laser at 532 nm at final MTX concentration of 0.1 mM. Significant reduction of cell viability was recorded due to heat generated by the plasmonic effect of AuNPs, which disassembled the IC causing MTX release.

The use of light radiation to kill tumor cells by the action of heating of a substance is termed as photothermal therapy (PTT). Lu et al. (2018) synthesized spherical covellite (CuS) NPs of around 18 nm, which depicted strong near infrared (NIR) wavelength absorption making it potent cancer therapy. Photothermal efficiency of CuS was recorded to be 60% higher than Au nanorods in this study. When the NPs were irradiated with 980 nm laser of 1.5 W/cm^2^ for 4 min it led to increased death of HeLa cells. The CuS NPs had abundant carboxyl groups, which were used to link amino modified β-CD and adamantine-modified arginine–glycine–aspartic acid (RGD) peptide inclusion complex. Furthermore, DOX could be loaded on nanoparticles by hydrophobic and electrostatic attraction. Drug release studies were performed to test the suitability of the NPs as a drug carrier at pH 7.4 and 5. Much faster release was seen at pH 5 due to protonation of -COOH groups and increased solubility of DOX at lower pH. MTT assay conducted on HeLa cells using CuS-DOX showed good inhibitory effect at 10 μg/ml concentration and the best inhibition rate when exposed to NIR. The RGD modification increased the cellular uptake of NPs due to its targeting ability toward integrin-positive HeLa cells.

Another group synthesized NIR light responsive nanocontainers consisting of mesoporous silica (mSiO_2_) and Tm, Yb, and Y metal core capped with CD-DOX ICs. The metallic NPs were shelled with mSiO_2_ *via* sonication, and the functionalization of stalks was carried out by the chemical method. Thereafter, DOX and α-CD were loaded *via* incubation stirring. HRTEM readings gave spherical configuration of the formed NPs whose size were around 50 nm (30 nm core +20 nm shell). NIR irradiation triggered the drug release and was confirmed by *in vitro* studies as 81% cumulative release was observed at 1.4 W/cm^2^, 64.2% at 1 W/cm^2^, and 41.1% at 0.4 W/cm^2^ of 980 nm laser light. HeLa cell line-based study gave insights that nanoparticles in the presence of NIR irradiation were not harmful to the cells until DOX was added. Increasing DOX concentration with higher radiation strength gave better growth inhibitory action at 50 μg/ml with 1.4 W/cm^2^ (Cui et al., 2015).

An important requirement in the drug delivery is its ability to reach remote tissues and exhibit desired activity over a defined period of time. With the growth of research involving magnetic NPs, this is very much possible. One of the many works involving magnetic NPs, Hariharan et al. (2019) used samarium ferrite with a coating of CD-fluorouracil. The size of NPs formed was 52 nm with spherical configuration. The drug release studies were conducted at 35° and 40°C simultaneously in pH 6 and 7.4, respectively. Faster drug release was seen at pH 6.0 at hyperthermia-like conditions (40°C). The quicker release of the drug can be attributed to acid-responsive nature of cyclodextrins. At 10 μg/ml there was enhanced cytotoxicity in HeLa cells as determined by MTT assay.

Another MNP-based work was conducted using β-CD-ethylene diamine tetra acetic acid (EDTA) and iron oxide nanocarrier. SEM analysis indicated that the nanocarrier were spherical, agglomerated with a porous network structure. Encapsulation efficiency of CPT in the nanocarriers was recorded to be 87%. The dialysis technique was used to assess the drug release profile at pH 2.4, 5.5, and 7.0 at 37°C. Nearly 65, 61, and 58% of CPT were released after 10 h at pH 2.4, 5.5, and 7.0, respectively. IC_50_ of the drug containing nanocarrier was 245 μg/ml against HeLa cells as determined by MTT. Phase contrast microscopy and acridine orange/propidium iodide (AO/PI) staining assay detected the membrane blebbing and nuclear fragmentation, which are the signs of apoptosis. Furthermore, annexin V/PI (flow cytometer assay) confirmed early and late stage apoptosis. Also other studies indicated change in mitochondrial membrane potential, higher caspase 3 expression, and cell cycle arrest at G2/M phase, which all collectively contribute to the death of cervical tumor cells (Rajan et al., 2017).

Lanthanide ions have the ability to absorb NIR light and convert them to higher energy photons effectively. Hence, Wang et al., 2016 demonstrated the drug delivery function of upconversion NPs (UCNP) using self-assembled structure of lanthanides, COOH-β-CD with inclusion of adamantine phthalocyanine. The size of the roughly spherical structure was about 85 nm as measured by TEM. UCNP-mediated PDT is a FRET process, therefore it needs photosensitizer to be close enough to UCNP. This was achieved through the formation of the nano-assembly to maintain the FRET efficacy. Whether the activity of the UCNPs inside the cell is present was checked by singlet oxygen sensor green assay for ^1^O_2_ generation. UCNP + Ad-ZnPc and UCNP/COOH-β-CD/Ad-ZnPc exhibited obvious fluorescence intensity inside HeLa cells cytoplasm indicating its effective internalization and reactive oxygen species (ROS) generation. The measurement of anticancer activity inside cells was assessed by trypan blue assay, which indicated that the cancerous cells having UCNP + Ad-ZnPc mixture and UCNP/COOH-β-CD/Ad-ZnPc were dead. Also, the PDT effect was measured after illumination of 980 nm laser using MTT assay, which gave dramatic decrease in cell viability depending on dose and light.

Recently, circular RNA emerged as a biomarker for cervical cancer and shows tissue specific expression. They play an important part in the progression of tumors by regulating several microRNAs (Chaichian et al., 2020). Hence, such novel biomarkers and targets should be studied and employed for diagnosis and therapeutic interventions. The usage of gene/RNA-based therapies in combination with other therapies is of value as they increase the treatment efficiency. The photodynamic approach can be used in cases where chemotherapy does not work effectively. Hence, different kinds of photosensitizers and nanoparticles to carry them need to be worked on.

### 4.3 Liver cancer

Liver due to its high metabolic activity tends to build up multiple ROS, which can be another reason for inflammation and cancer other than genetic factors and exposure to foreign substances (Liou and Storz, 2010). Liver cancer has a high incidence rate and is responsible for over 7 lakh deaths every year all around the world (Liver Cancer Statistics, 2021).

Hence, researchers have tried to build magnetic nanoparticles for combined chemotherapy (CT) and photothermal therapy (PTT) using doxorubicin as model drug whereas polydopamine (PDA) as a photothermal agent. PDA is non-toxic with strong adhesive properties and can be combined with both polar and non-polar surfaces. The resulting nanoparticles were having spherical diameter in range of 8–14 ± 2 nm and the PDA layer to be around 2–3 nm thick. The zeta potential was measured to be −27 mV. The LC was determined to be ∼900 μg/mg with 90% EE. The cumulative release studied at pH 4.5 and 5.5 was seen to give slow and sustained release up to 50 h giving results of 9 and 10%. When the cancer cells were irradiated using 808 nm laser beam with 2 W/cm^2^ for 5 min, stronger PTT effect was observed for nanoparticle concentrations above 5 μg/ml (around 60% cell viability decrease). It was concluded that simultaneous application of CT-PTT by using Fe_3_O_4_@PDA@SH-β-CD at 5 μg/ml gives a better therapeutic result (Mrówczyński et al., 2018).

Another photo-chemotherapy (PCT) method carrying CPT as drug and magnetic graphene oxide (MGO) as photothermal agent was prepared *via* EDCI/NHS condensation followed by the freeze drying method (Wen et al., 2021). The MGO containing Fe_3_O_4_ were conjugated with β-CD-cholic acid-hyaluronic acid (CCH) polymers for enhanced targeting toward hepatic cells. The MGO/CCH nanocomplex had high photothermal conversion efficiency at NIR wavelengths (808 nm) such that their temperature could reach until 71°C, with short laser irradiations. Furthermore, the NIR irradiation stimulated the release of CPT drug from the nanoconjugates. *In vitro* study to evaluate combined cytotoxicity of the PCT method was carried out upon BEL-7402 (human hepatoma cells) K-150 (human esophageal cancer cells), HCT-116 (human colon cancer cells), and HL-7702 (human hepatocyte cells). The highest mortality was observed in the BEL-7402 illustrating the targeting ability of the MGO/CCH nanocomplex. Furthermore, studies *in vivo* of BEL-7402 tumor containing nude mice exhibited strong tumor inhibition rate (>90%), showing the superiority of the synergistic therapy. Application of such targeting agents with dual mode of treatment enhances the therapeutic efficacy of the nanotherapy systems.

Another similar work involved dual action of photodynamic and photothermal therapy utilizing Fe_3_O_4_@Cu_2-x_S as the core upon which α-CD-Ce6 was coated *via* self-assembly. Singlet oxygen was generated by Ce6 upon visible light irradiation, especially at 525 nm wavelength. The coordinated interaction of Cu with Ce6 further enhanced the ^1^O_2_ generation. Furthermore, it was observed that unlike other agents this nanocomplex has a photothermal effect at the irradiation of NIR-II laser (960 nm). The maximum temperature reached by 100 μg/ml of the nanoparticles under this condition was 48.1°C. MTT assay upon HepG2 cells under visible + NIR light resulted in high cytotoxicity of the NPs. Further assays indicated the apoptosis mode of cell death in the cancer cells elicited by the nanocomplex. Efforts to design multifunctional components in the quest to fight cancer can help us to develop an effective therapy (Zhang et al., 2021).

Chen et al. (2016) designed disulphide bridged β-CD in conjugation with adamantane containing hyaluronic acid polymer (HAD-β-CD) loaded with PTX drug. The hydrodynamic diameter of the formed nanocomposite was around 231 nm. Also, the EE and LC were 32 and 3%, respectively. In PBS, the free PTX dissolved and reached equilibrium in about 2 h; however, the HAD-β-CD assembly held the drug strongly did not release them until DTT or hyaluronidase was added to the solution. This dual stimuli property can be exploited to achieve targeted drug release efficiently. Moreover, MTT assays showed the IC_50_ of the nanoformulation was comparable to the free MTX drug, that is, around 3.2 μg/ml. The authors state that the endogenous glutathione and hyaluronidase in the cell cytosol helped in the breakage of the hyaluronic acid assembly and facile release of drug.

Similarly, Fan et al., 2019 prepared folic acid–polyethylene glycol-β-CD (FA-PEG-β-CD) NPs to carry DOX. CD increased the carrying capacity of the drug whereas, PEG helped in prolonging the residence time of the API in the bloodstream. The NPs had 55 nm diameter and an excellent EE of 95.2% with LC of 11.9%. Also, its drug release properties were faster in a more acidic pH of 5.5 than the bodily pH of 7.2. Furthermore, hemolysis percentage was less than 5% in concentrations up to 100 μg/ml of blank NPs. CCK-8 assays resulted in less than 25% cell viability of HepG2 cell line at 100 μg/ml DOX-loaded NPs within 24 h.

Chen et al. (2020) developed a β-cyclodextrin-modified Pt (II) metallacycle-based polymer for nitric oxide (NO) responsive DOX delivery. Metallacycles are those carbocyclic compounds where at least one carbon is substituted with a metal. The DOX drug loading amount was 23.95%. The formed nanopolymers have tendency to self-assemble, and the average spherical diameter of them was observed to be roughly 300 nm in both SEM and TEM. The NO responsiveness was verified morphologically using TEM studies. When excess of NO was added to the assembled globular NPs, smaller strips of aggregates were observed likely due to cleavage of the amide-functionalized o-phenylenediamine units. Prolonged incubation time led to complete disassembly of the sphere. Therefore, *in vitro* drug release study was conducted both in the presence and absence of 50 μM NO. As expected around 58% of total drug was released in 48 h; however, in the absence of NO only 15% of the drug released. This confirms the targeted release property of the drug delivery system (DDS) since it is known that higher levels of NO and oxidative stress are seen in liver cancer. MTT assay over the period of 24 h performed against HepG2 cells using nano-formulated DOX exhibited higher cytotoxicity at all concentrations when compared to free DOX. These observations indicate that combination chemotherapy systems can improve cancer treatment.

Drugs like docetaxel have activity against wide types of carcinoma and have been approved for the treatment of prostate, breast, and lung cancer. However, their usage is limited due to its systemic toxicity and low solubility. Modified cyclodextrins were used by Xu et al. (2016) to mitigate this problem. The folic acid–CD complex was biocompatible and found to be non-toxic to normal KB cell line. Flow cytometer studies indicated that folic acid (FA)-targeted CDs were accumulated the highest in KB cells followed by HeLa cell lines. Folate receptor (FR) negative HepG2 cells had significantly lesser build up. Time-dependent studies implied that FA-targeted complex is taken up *via* endocytosis of FR positive cells. Also FA competition assays confirmed the affinity of the nanocomposite toward the FR positive KB cells, therefore it was established that the uptake of nanoparticles is mediated through FR mediated endocytosis. Apoptosis studies were also conducted, which gives the result of 38.75, 64.6, and 99.1% for the HepG2, HeLa, and KB cells, respectively. Mice studies showed that more of the drug retain in tumor sites instead of other organs at 4 h due to the efficient targeting capacity of the drug carrier.

Gambogic acid (GA) is known to downregulate telomerase activity to achieve the anticancer effect. But the poor hydrophilicity and systemic circulation prevents it from clinical development. Magnetic nanoparticles (MNPs) have an ability to respond to external magnetic field such that it can be moved and concentrated at the target site thus increasing the drug availability. For this, effort was taken to amalgamate GA-β-CD inclusion complex to MNPs *via* co-precipitation method. Observing the NPs in HRTEM showed spherical core-shell like structure. Zetasizer confirmed the size of NPs to be approximately 147 nm with +29.3 mV zeta potential. The EE and LC were found to be 85.71 and 4.63%, respectively. Drug release studies at pH 7.4 resulted in 30% release within 100 min whereas release was lower at pH 4.0. IC_50_ values were 0.348 and 0.964 mg/ml for HL-60 and HepG2 cells, respectively. The amount of released GA over 12 h was approximately 67% in the free GA group *in vitro*, while the half-life was approximately 6.9 min after intravenous (IV) administration to the rats. The amount of released GA over 12 h was 75% in the Fe_3_O_4_@NH_2_-β-CD@GA MNPs group *in vitro*, while the half-life was approximately 18.8 min after IV administration to rats *in vivo.* It is anticipated that the sustained release and improved half-life of the drug will have a check on the growth of other hidden cancer cells too, which may be resistant (Fang et al., 2019).

An approach to increase drug content in the nanoparticles using barbigerone (BAB) as model is given by Qiu et al. (2015). They first formed BAB-HP-β-CD inclusion complexes (ICs) and then encapsulated them in liposomes. By altering the ratios, they could achieve 60.78% EE with an average diameter of about 60 nm as observed under TEM. *In vitro* release of BAB ICs in liposomes was slow and sustained unlike the plain inclusion complexes, which leaked around 80% drug in the first hour itself. In addition, they were non-hemolytic like saline. Decrease in cell viability of HepG2 cells were seen through MTT assay after the addition of 20 µM concentrations of liposome drug inclusion complex. Moreover, HepG2 tumor cell-bearing nude mice were studied for the efficacy of the liposome-based drug delivery system. The average survival time of the treated mice was a significant 67.3 days as compared to 45.3 days for control group mice.

Zheng et al. (2021) engineered a pH-sensitive nanoparticulate system using acetalated CD, zinc phthalocyanines, and PEG to deliver doxycycline (ZnPc-(PEG)_5_:Ac-CD:DOY). The spherical diameter of DDS was measured to be 229 nm using DLS and TEM. The zeta potential was −23 mV, which means they have good colloidal stability. Flow cytometry results indicated that the drug released from NPs elicit apoptosis and necrosis. Whereas fluorescence microscopy studies after photothermal treatment (680 nm, 1.5 J/cm^2^) indicated that they damage the mitochondria. MTT assay conducted on HepG2 cells along with illumination of 50 μM of ZnPc-(PEG)_5_:Ac-CD:DOY led to only 2.1% survival rate of the cells. Furthermore, H22 tumor-bearing mice were injected with 0.2 μmol/kg of ZnPc-(PEG)_5_:Ac-CD:DOY and illuminated with 680 nm light source (3 min, 50 J/cm^2^) for 7 days. The synergistic cytotoxic activity of chemotherapy and photothermal therapy led to smallest tumor size of all groups due to better tumor targeting as shown in the 3D fluorescence molecular tomography. The harvested organs did not show any signs of pathological changes, signifying the safety of the DDS. Similar multifunctional DDSs can improve both drug delivery and treatment efficacy.

Due to the presence of multiple pathways and its varied functions, liver cancer is harder to diagnose. Newer and specific biomarkers need to be discovered for the earlier prognosis and effective treatment of liver cancer. MicroRNAs are a possible disease marker and need further studies (Tunissiolli et al., 2017). Researchers can also try to use the high rate and variety of metabolic functions taking place in the liver to their advantage and exploit them for specific drug delivery. Apart from pH, GSH, and FR other targets can be chosen for the purpose.

### 4.4 Lung cancer

Non-small cell lung cancer represents around 80% of all lung cancers and is the leading cause of cancer deaths globally (Siegel et al., 2020). Even though breast cancer is most frequently diagnosed, it is easier to cure, making lung cancer the deadliest of the cancers. Many epidemiological reasons like cigarette smoking, exposure to pollution, familial inheritance, and mutations can cause lung cancer (Araujo et al., 2020).

Erlotinib (ERL) is a potent and selective tyrosine kinase inhibitor, which can stop auto phosphorylation and result in apoptosis induction. But it lacks the ability to specifically target cancer cells and causes many side effects such as intestinal disorders, diarrhea, and skin rashes. In a study, effort was taken by Vaidya et al. (2019) to increase the entrapment efficiency of the drug by forming drug-CD complex and enclosing them into PLGA (ERL-CD-PLGA) for controlled release. The EE increased to 61.5 ± 3.2% inside the 210 ± 8 nm spherical NPs whereas LC was ∼5% as the drug solubility increased from around 1 mg/ml to 7 mg/ml at 4% SBE-β-CD concentration. *In vitro* release studies in 7.4 pH at 37°C demonstrated highly controlled release of drug as only 32 ± 4% of encapsulated drug was released in 5 days. Both, erlotinib resistant and sensitive lung cancer cell lines were used to understand the impact of the formulation. IC_50_ of A549, H517, H460 (ERL-resistant), and H4006 (ERL-sensitive) cells were recorded to be 4.5, 11.1, 5.5, and 0.21 µM, respectively against ERL-CD-PLGA NPs. At 5 µM concentration of NPs the viability of A549 cells was around 32% as compared to the 73% viability of free drug treated cells. This improved cytotoxicity is hypothesized due to increased intracellular uptake and slow release of drug. Further apoptotic studies performed using flow cytometry confirmed the increase of DNA fragmentation in nano erlotinib treated cells. Furthermore, CaspaseGlo 3/7 assay confirmed the increase of caspase-3 and caspase-7 expressions. Cleaved PARP was observed in the Western blot analysis, which can also be responsible to induce apoptotic cascade. 3D spheroid cultures were also used to evaluate the tumor reduction efficiency of the nano erlotinib, which showed 1.5–2-fold more efficiency in tumor volume reduction as compared to plain drug solution, which was attributed to the better penetration of the NPs into the tumor microenvironment.

The same group took a synergistic approach to treat non-small cell lung cancer by combining the ERL-CD-PLGA NPs with another nanocomplex containing quinacrine (QA). QA is an approved drug, which also possess tumor suppressing activity *via* p53 gene and autophagy modulation. The ERL NPs was formulated with QA containing PEI-PLGA NPs *via* sonicated aqueous emulsion method. Based on combination studies, the pre-incubation of QA for 6 h before the introduction of ERL to A549 cells elicited higher cytotoxic effect. The same was studied in 3D spheroid cultures of A549 cells over a period of 15 days, which resulted in an observation of smallest volume of tumor size. The combined synergistic action of QA-ERL NPs was further validated when molecular markers like caspase-3 expressed indicating increased apoptosis rate (Kulkarni et al., 2021). The evidence on synergetic approach to treat cancer are ever increasing and this methodology has a great translational capability into a clinical treatment.

A green chemistry approach was taken by Alhakamy et al. (2022) to develop a pumpkin oil (PO)-based nanoemulsion carrying costunolide (CTD) as anticancer drug. The α-CD-PO-CTD nanoemulsion complex was synthesized using the high shear homogenization method. The nanovesicles formed were having diameter of ∼196 nm. Cytotoxic MTT assay upon A549 cells using nano-CTD yielded an IC_50_ of 6.1 µM. Green approaches like this are both innovative and at the same time shall minimize the usage of synthetic surfactants, which could be toxic. Steps to design the novel drug delivery system utilizing the natural resources are highly sustainable.

In effort to develop a suitable carrier optimizing the delivery of DOX, NPs were prepared using charged PGA-β-CD and PGA-γ-CD with a polypeptide backbone. The formed NPs show hydrodynamic diameters of around 5.5 nm as per DLS measurements. PGA-γ-CD depicted more negative zeta potential (-47 mV) than PGA-β-CD (−32 mV) at pH 7.4. It is worth noting that zeta potential of both these polymers varies as a function of pH. At pH 2.5, the zeta potential of PGA-β-CD and PGA-γ-CD is −6 and −4 mV, respectively. In the interval of pH 9–13, both systems have a more negatively charged surface with zeta potential ranging from −48 mV to −69 mV. Since, at a lower pH the zeta potential decreases the nanoparticles tend to accumulate and sustain in the tumor environment to elicit damage to the cancerous cells. MTT assay was performed on A549 cells, in which the IC_50_ of PGA-β-CD and PGA-γ-CD was 116.7 ± 20.7 and 88.2 ± 30.7 nM, respectively (Oliveri et al., 2017).

Zhang et al. (2016) were interested in reporting the pharmacological activity of CD-complexed curcumin NPs. MTT assay showed increased activity in a concentration-dependent manner. Higher CUR concentration (∼55 µM) was needed to achieve IC_50_ in A549, H446, and H520 lung cancer cells whereas nanoparticulate CUR showed similar activity in a much lower concentration (∼10 µM). Hoechst staining detected the induction of apoptosis in A549 cells after 24 h. The expression of CyclinE and CDK2 was noted to be decreased, while the p21 and p53 levels increased which indicates the G1-phase arrest of cells, which could initiate apoptosis. Also levels of Bax and caspase-3 proteins were seen to be increased, which also play roles in the apoptotic pathway.

Another group conjugated all three (α-, β-, and γ-) types of CD-CUR complexes to PEGylated AuNPs. The gold core was sized 5 nm, and the total diameter of the spherical nanoconjugate was less than 30 nm as analyzed by TEM. Phase solubility and UV–Vis studies was conducted to reveal that beta cyclodextrin had the best carrying capacity of the drug with an LC of 13.1%. However, all three types of cyclodextrin nanoconjugates exhibited significant cytotoxic effects on A549 cells similar or better than free CUR at 50 µM concentrations. Furthermore, studies on intracellular activity of the nanoconjugate using coumarin-6 fluorescence indicated the uptake of them by endocytosis and their localization in endosome (Hoshikawa et al., 2018). PEGs both improve solubility and intracellular uptake of NPs.

SPIONs were chosen by Santos et al. (2018) to functionalize with β-CD-DOX ICs. The iron core was roughly 14 nm while the whole structure had a hydrodynamic diameter of 185 nm. The zeta potential was found to be −28 mV. Inclusion of the CDs increased the EE up to 81%. The drug release conducted in absence of stimulant (alternating magnetic field) was less than 45% in both pH 5.0 and 7.4 at a temperature of 45°C after 50 min. But in its presence the DOX release increased to 92 and 85% at pH 5.0 and 7.4, respectively. This accelerated release was related to the Neel and Brown relaxation processes due to magnetic action. Furthermore, Presto blue viability assay was conducted on A549 cells, which in presence of the NPs showed reduction in cell viability.

A multifunctional nanocontainer was created using iron oxide core-mesoporous silica shell functionalized with cyclodextrins and PEG. DOX was loaded onto the nano-sized silica pores where CDs contained them as gatekeepers. The central core was around 22 nm making the total diameter of the NPs 80 nm. The PEGylation prevented the precipitation of NPs. Experiments showed that DOX does not release in the dissolution medium until at least 0.01 mM glutathione was added to the medium. This indicated the CD gatekeepers are firmly tied to the silica surface preventing uncontrolled drug release. Cytotoxicity and terminal deoxynucleotidyl transferase dUTP nick-end labeling (TUNEL) assays confirmed the apoptotic death of A549 cells in the presence of 1 and 2 µM NPs. Also, their MRI capability was reported due to significant reduction of transverse relaxation time compared to the control. Within 2 h of injection of NPs in A549 xenograft mice, the tumor region turned into red. This signal intensity increased for 1 week after which it gradually turned into green due to removal of the NPs. This also led to the increase of tumor size after 2 weeks as the DOX availability was reduced (Lee et al., 2012).

Resveratrol (RES) is one of the famous polyphenols, which possess anti-aging, antitumor, and antioxidant properties. Nevertheless, like other natural compounds it is poorly bioavailable in aqueous medium. Wang et al. (2020) complexed sulfobutylether-β-cyclodextrin with RES and encapsulated them into PLGA thereby creating an inhalable formula. TEM was used to visualize the ∼264 nm spherical polymeric NPs. The RES containing polymeric NPs had an EE of 29% and LC of 0.72%. The CD-RES NPs was seen to be internalized into A549 cells within 3 h of incubation by fluorescence microscopy. The IC_50_ values for CD-RES NPs and RES studied using MTT assay were found to be 3.3 ± 1.4 μM, and 50.8 ± 10.0 μM against A549 cells. Similarly when evaluated against H358 cells (non-small cell lung cancer cell line), the IC_50_ values were found to be 1.0 ± 0.5 μM, and 50.0 ± 19.1 μM. Moreover, clonogenic assay specified that the nanoparticulate RES reduces the probability of tumor relapse better than free RES. Other than that, results of scratch assay favored the inhibition of metastatic capacity of A549 cells greatly. The study was comprehensive after the CD-Res NPs exhibited 1.3–2.4-fold reduction in the volume of 3D tumor spheroids of A549 cells at both single and multiple doses. Other mechanistic studies indicated that RES induces apoptosis in the cancerous cells *via* caspase-3.

A recent study strived to improve the targeted inhibition of lung cancer. The authors functionalized cubic γ-CD metal organic framework with GRGDS pentapeptide and loaded low molecular weight heparin along with DOX (RCMLD). The SEM and DLS results revealed that the RCMLD had a cubical morphology with an average size of 150 nm and zeta potential of −25 mV. The unloaded nanoscale drug delivery system was non-toxic to A549 cells, B16F10 melanoma cells, and WI26-VA4 normal alveolar epithelial cells up to 133 μg/ml. The *in vitro* release of loaded DOX reached almost 80% in presence of 10 mM GSH, whereas in its absence only 40% was achieved over the period of 72 h. When RCMLD was injected to B16F10 metastatic lung cancer-bearing mice it accumulated the most in lung tumors, almost six times greater than the liver. Further *in vivo* studies involving A549 tumor bearing mice was performed where RCLMD (DOX at 1.0 mg/kg) was injected at an interval of 7 days. The size of the tumor progressively decreased, and almost negligible metastasis was observed. Similar results were obtained in B16F10 tumor bearing mice. Moreover, the administered mice did not show any symbols of pulmonary distress or histopathological changes except those due to the drug (He et al., 2021).

Multiple methods for detecting lung cancer are underway taking into consideration breath analysis. However, proteins like carcinoembryonic antigen (CEA) and vascular endothelial growth factor (VEGF) are currently established (Roointan et al., 2019). More studies in this direction will be appreciated because it is one of the most difficult cancers to treat. Nonetheless, efforts could be taken to reduce the size of nanoparticles delivered for better penetration into lung tissues. Newer effective anticancer drugs can be selected for development.

### 4.5 Prostate cancer

Almost two lakhs prostate cancer are expected to befall in the United States alone (Siegel et al., 2020). Similar to other cancers, this neoplasm can be triggered by genetic changes, cigarette smoking, and other lifestyle habits (Rawla, 2019). Advancements in diagnosis and treatment along with increased screening have helped in decreasing the mortality rate of men due to prostate cancer (Litwin and Tan, 2017). Cyclodextrin-based polymers gained importance as early as 2000s to perform gene therapy as an option for cancer treatment (Hwang et al., 2001). This section expands the different instances where cyclodextrin nanoparticles show potential to combat prostate cancer.

Ntoutoume et al. (2016) checked the delivery efficiency of CUR using CD-containing cellulose nanocrystals. Cationic cyclodextrins were complexed with anionic cellulose to form rod-like nanocrystals for carrying CUR. The hydrodynamic size of the NPs was 206.8 nm, with a zeta potential of −29.6 ± 2.7 mV and 10% LC. The *in vitro* release of CUR after 8 h was 24%. Higher amounts of CUR were seen to be accumulated in the cells treated with nanoformulation. This shows that cellulose nanocrystals have a vital role in increasing the cell uptake. The IC_50_ values calculated against PC3, DU145, and HT29 cells after 48 h were 7.5, 5.5, and 4.5 µM, respectively. These confocal microscopy and antiproliferative assays suggested the mechanism by which the nanoconjugate acts is by internalization through endocytosis and followed drug release.

Tanshinone (TAN) and α-mangostin (MAN), drugs known in southern Asia used in ancient pharmacopoeia has proven its cytotoxicity against various cancer cell lines. However, their clinical use is hampered by its short half-life and poor solubility. The high versatility of gold nanoparticles allows the adherence of cyclodextrin inclusion complexes upon its surface *via* polymers like polyethyleneimine (PEI). PEI is cationic and can deliver functional molecules inside the cells by passing through the cell membrane. Therefore, these small composites could target the tumor tissues by the EPR effect. The hydrodynamic diameter of the nanocomplex was around 100 nm as per DLS and UV spectroscopy measurements. The drug LC for TAN and MAN were 1.3 and 4.6%, respectively. *In vitro* release studies without stirring showed that 80% of TAN released after 5 h. 6 µM of TAN was required to show IC_50_ for both DU145 and PC3 cells, whereas 6 and 17.5 µM of MAN was required to achieve 50% cell death for DU145 and PC3 cells, respectively. The cell death pathway was identified using enzyme-linked immunosorbent assay (ELISA) quantification of nucleosomes in the cell lysates. The data obtained showed they implicit DNA fragmentation, which confirmed the apoptosis-mediated cell destruction (Qiu et al., 2016).

The amphiphilic nature of cyclodextrins was exploited to synthesize “giant surfactants” in cohesion with calixarene rings. Calixarene is attached with hydrophobic chains such that after self-assembly, CDs face outside while covalently linked calixarene module to the core. Thus obtained EE and LC were outstandingly high, 98 and 74%, respectively. The drug DTX was loaded in the core matrix formed by the nanospheres and nanocapsules. Cryo-TEM micrographs indicated quasi-spherical structures with size around 35 nm for nanospheres and 150 nm for nanocapsules. The *in vitro* drug release profile had an initial burst for 6–8 h and sustained release up to 60 h. Lactate dehydrogenase (LDH) assay results supported the anticancer activity of the drug delivered by the nanoassemblies in human prostate cancer cell line. Almost 80% of LDH was released in LNCaP cell line when treated with the nanocomplex (Gallego-Yerga et al., 2017).

Same group further took the approach of synthesizing self-assembled amphiphiles using modified cyclodextrin and calixarene moieties for encapsulating the drug DTX. The methylated-β-cyclodextrin and calixarene groups were linked with thiourea-based linker, which has the property of redox sensitivity. This improved the drug release efficiency since tumor cells contain higher concentrations of GSH concentrations. As well as the drug encapsulation was higher due to their amphiphilic nature and large cavity formation *via* nano-scale interactions. The components were seen to be assembled either as nanocapsules or nanospheres, which had a hydrodynamic diameter of 40–100 nm due to aggregation of smaller NPs ranging from 10 to 40 nm. Cryo-SEM confirmed the spherical shape and ∼100 nm size of the nanoformulations. Docetaxel encapsulated in the nanospheres released in a rapid rate even after the burst release and reached 100% within 24 h in the presence of GSH. However, in its absence the rate of release was much slower and reached 100% only after 72 h. Similar trends were seen for nanocapsules also. The IC_50_ values for DTX-encapsulated nanospheres and nanocapsules obtained for LNCaP and PC-3 prostate cancer cell lines were 0.9, 1.1, 1.3 nM, respectively, whereas the free drugs had a much larger IC_50_ values of 15 and 10 nM, respectively. The cytotoxic activity is deduced to be the apoptosis initiation and mitosis arrest exhibited by the GSH-triggered release of DTX (Gallego-Yerga et al., 2021).

RNA interference (RNAi) has been considered as a potential treatment for many diseases including cancer. Evans et al. (2017) studied the folate targeted delivery of CD with fusogenic peptide glycine–alanine–lysine–alanine (GALA), which can facilitate improved endosomal escape. The formation of inclusion complex was confirmed as the gel showed no siRNA band when formulation was loaded in it. Also the stability of PEGylated NPs in salty media was checked, which maintained the spherical shape and size of 200 nm, which was reported from TEM. The complexes then formed were used to deliver ZEB1 and NRP1 siRNA into prostate cancer cells. Importantly, the presence of CD and PEG protected the siRNA from serum nucleases as long as 8 h as tested in 10% FBS. Competitive uptake studies using excess free folate reduced the nano formulated compound’s uptake into cells. Luciferase reporter gene assay was conducted in PC3Luc cells, and the knockdown was measured; 55.91 ± 6.96% reduction was seen for folate-containing formulation, whereas adding GALA led to increased knockdown of 76.99 ± 10.89%. Desirably, the genes should be silenced both at mRNA levels and protein levels, which was confirmed by densitometry, RT-PCR, and Western blot analysis. There were approximately 50 and 60% reduction in ZEB1 and NRp1 protein levels, respectively; 3D PC3 spheroids cultures were incubated with the folate-GALA formulations, which showed more reduced and compact colonies compared to the controls.

Nanosponges are known for a large holding capacity, one such study evaluated CPT-β-CD nanosponges with a prolonged release profile and no burst effects. Multiple therapeutic studies including cell adhesion, migration, angiogenesis, and STAT3 phosphorylation was conducted. The spherical 400 nm nanosponges inhibited all of them better than free CPT in PC-3, DU145, and HUVEC cells. The inhibitory effects inflicted by them demonstrate that nanosponges can prevent and reduce metastatic effects. Moreover, *in vivo* studies on SCID mice with PC-3 cells when subjected to 2.5 mg/kg of CPT-β-CD nanosponges inhibit tumor growth and vascularization within them. As well, the nanosponges did not affect body weight, motor activity, and feeding behavior (Gigliotti et al., 2016).

Another β-CD-based nanosponges linked by 1,1′-carbonyldiimidazole (CDI) was formulated using the freeze-drying method to load the drug flutamide (FLT). FLT works by targeting the androgen receptors for treating prostate cancer. The nanosponges sized around 99 nm as per DLS. Due to increased wettability of the nano FLT in complexation with CD enhanced the drug releasability and showed complete release within 180 min. Rhodamine B labeling assay showed the internalization of the nanosponges into PC3 cells with high fluorescence intensity at 3 h. However, the MTT assay conducted showed similar or lower cytotoxicity of nano FLT in comparison to plain FLT due to higher accessibility of the latter and slower release of the former (Allahyari et al., 2021).

A ternary nanoassembly was formulated by Zagami et al. (2019) incorporating pheophorbide, a photosensitizer. Adamantanyl–folic acid conjugate was used for targeting alpha-folate receptor overexpressing cancer cells, while modified CD stabilized the structure. The spherical NPs were sized between 200–300 nm having −50 mV zeta potential. Uptake of the NPs is markedly different in MCF-7 cells and PC-3 cells because breast cancer cells have higher expression of folate receptors. Although the nanoparticles are effective against both the cell lines with IC_50_ of 270 and 700 nM, respectively, there is an enormous difference in the concentration. The phototoxicity was activated when 0.9 J/cm^2^ red light emitting diode (LED) light was shined on the NP incubated cells. The apoptotic effects were also measured greater in MCF-7 cells than PC-3 cells *via* annexin−propidium iodide assay. The authors further suggest that conducting a pre-hand investigation on the molecular components responsible for cell death mechanisms can be helpful in developing strategies to prepare successful multifunctional nanosystems.

Wu et al. (2020) studied various nanosystems for analyzing the delivery and cytotoxicity of β-lapachone (β-Lap) on prostate cancer cell line. The apoptotic and cytotoxic activity of the drug is achieved when the cytoplasmic enzyme NAD(*p*)H:quinone oxidoreductase (NQO1) reduces β-Lap. This is pertinent because numerous studies reported the overexpression of this enzyme in several cancers including prostate adenocarcinoma. HP-β-CD, SBE-β-CD, liposome/HP-β-CD, and liposome/SBE-β-CD among others were studied for its encapsulation efficiency and *in vitro* cytotoxicity. The encapsulation efficiency calculated for liposome/HP-β-CD and liposome/SBE-β-CD were approximately 24.36 and 27.98%, respectively. The MTT cytotoxicity assay was performed for the duration of 48 h to obtain the IC_50_ values exhibited by the drug delivery systems, which were 1.6, 1.7, 1.8, 1.9, and 2.2 µM for free β-Lap, HP-β-CD, SBE-β-CD, liposome/HP-β-CD, and liposome/SBE-β-CD, respectively. But similar concentrations of drug inhibited the growth of PNT2 cells, normal human prostate epithelium cells. Both liposome containing CD–drug complex and CD–drug inclusion complexes presented good capacity to solubilize the drug and deliver it; however, the authors concluded that better results can be achieved with ligand functionalization and active targeting.

Another FA-functionalized NPs were prepared based on β-CD conjugated quaternary QDs (Ag, In, Zn, and S) to carry an unsymmetrical bisacridine drug C-2028. The nanocarrier was prepared using simple 4-dimethylaminopyridine mediated co-preparation method and dialysis. C-2028 was complexed into CDs as ICs by overnight stirring incubation. The amount of drug loaded on 1 g of nanocarriers was around 6.16 mg. The nanocarriers without C-2808 did not affect the growth of both cancerous and non-cancerous cells, which indicates their high biocompatibility. However in addition of nano C-2808 the Du-145 and LNCaP cells growth was inhibited and excellent IC_50_ values of 0.010 and 0.088 µM was observed, respectively. Also, CLSM studies suggested the multiple modes of endocytosis of the drug-nanocarrier including clathrin- and caveolae-mediated (Pilch et al., 2022). Increasing the biocompatibility of such drugs can aid scientists to repurpose known drugs into a potent anticancer drug.

Prostate-specific antigen is one of the long known markers of prostate cancer (Singh et al., 2019). However, conditions may vary from person to person and thereby concentrations too. Other known molecular targets such as epidermal growth factor receptor (EGFR), PARP, and VEGF are known but common for all cancers. Hence, there is a pressing need to find out novel and specific biomarkers/receptors for prostate and all other cancers to develop better and targeted release.

## 5 Clinical significance of cyclodextrin nanocomposites

Over the past 2 decades many nanoparticulate drug delivery systems have been under clinical trials for delivering diverse types of hydrophobic drugs and siRNA to treat human solid cancerous tumors (Hamaguchi et al., 2007; Plummer et al., 2011; Kato et al., 2012; Von Hoff et al., 2016). Cyclodextrin-based nanoparticles decorated with PEG (CALAA-01) were the first nanosystem carrying siRNA to reach the human clinical trials phase. Whereas products like MitoExtra containing mitomycin C complexed with HP-β-CD has successfully reached the market for treating multiple types of malignancies (Grosios, 2002). The nanocarrier delivered siRNA to target ribonucleotide reductase subunit 2 using transferrin as ligands. Biopsies of human tumors showed that gene silencing by the delivered siRNA was evident, and it did not show toxicity to the kidney and liver at administered doses (Davis, 2009). Another human trial study was conducted by NewLink Genetics Corporation, which reached phase II using alternating units of PEG and cyclodextrin. Phase I trials resulted in acceptable safety and improved circulation time owing to its pH-dependent release (Weiss et al., 2013). In the phase II trials, a reduction in the tumor size was seen in the patients with platinum resistant ovarian cancer and lung cancer (Pham et al., 2015). Furthermore, they evaluated advanced renal cell carcinoma using CRLX101 in a phase II trial, which was consistent with previous results but did not give newer safety signals (Voss et al., 2017). Recent studies focus on combination treatment of the cancer with NLG207 (formerly CRLX101) and enzalutamide which can overcome the drug resistance of enzalutamide *via* topoisomerase I inhibition mechanism (Schmidt et al., 2021).

A recent report from the clinical study of CRXL301 published that the taxane drug docetaxel had exhibited microtubule stabilization in metastatic castration-resistant prostate cancer patients. This was in positive correlation with the treatment time and reduces the number of CTCs in the blood (Mukhtar et al., 2020). Since cyclodextrin showed promising effects, currently clinical investigations for other cancer types such as metastatic castration resistant prostate cancer, urothelial carcinoma, and metastatic melanoma is ongoing as mentioned in Table 2 (Clinical Trials, 2022).

**TABLE 2:** Current and ongoing clinical trials for chemotherapy using cyclodextrin as carriers.

NCT number	Title	Condition	Intervention	Phase
NCT02055716	Sulforadex in healthy human males MAD	Prostate Cancer	Sulforadex, and alpha-cyclodextrin	I
NCT03531827	Combining CRLX101, a nanoparticle camptothecin, with enzalutamide in people with progressive metastatic castration resistant prostate cancer following prior enzalutamide treatment	Metastatic castration resistant prostate cancer	Enzalutamide, and CRLX101	I
NCT01948362	Sulforadex in healthy volunteers SAD	Prostate cancer	Sulforadex, and α-cyclodextrin	I
NCT00689065	Safety Study of CALAA-01 to Treat Solid Tumor Cancers	Cancer and solid tumor	CALAA-01	I
NCT00333502	Study of CRLX101 (NLG207) in the treatment of advanced solid tumors	Cancer and solid tumor	CPT conjugated to a linear and cyclodextrin-based polymer	I and II
NCT02769962	Trial of EP0057, a nanoparticle camptothecin with olaparib in people with relapsed/refractory small cell lung cancer	Urothelial cancer, small cell lung cancer, and prostate cancer	EP0057 and olaparib	I and II
NCT01803269	Topotecan hydrochloride or cyclodextrin-based polymer-camptothecin CRLX101 in treating patients with recurrent small cell lung cancer	Recurrent small cell lung cancer	Topotecan hydrochloride and cyclodextrin-based polymer-CPT CRLX101	II
NCT01612546	Pilot trial of CRLX101 in treatment of patients with advanced or metastatic stomach, gastroesophageal, or esophageal cancer that cannot be removed by surgery	Metastatic stomach, gastroesophageal, esophageal cancer	Cyclodextrin-based polymer-CPT CRLX101	II

## 6 Conclusion and future perspectives

Unfortunately, cancer is still responsible for the greatest number of deaths caused worldwide, second to only cardiovascular diseases. Tackling cancer in current situation heavily depends on chemotherapy. Hence, multiple novel drug delivery systems were developed with the conjunction of materials science, pharmaceutics, and biotechnology to carry various anticancer agents. The addition of nanotechnology to the cause could only improve the efficiency and effectiveness of such NDDSs. Nanoparticles can be tailored and functionalized to ensure the targeted delivery of variety of materials ranging anywhere from small chemical compounds to large biomolecules.

This is possible due to the development of nanosystems, which use supramolecular chemistry to render the nano-sized compositions relatively stable in systemic circulation and get triggered on the contact of tumor microenvironment. Bucket-shaped cyclodextrins are one such carbohydrate nano-oligomer, which has special physicochemical properties. We highlighted the role of cyclodextrins in this field emphasizing on its compatibility with wide variety of (bio)molecules due to its unique amphiphilic nature, toroidal shape, and supramolecular compatibility. The usage of cyclodextrin nanoparticles, especially improves the solubility, biocompatibility, and stability of hydrophobic anticancer drugs. In this article, we reviewed how *via* tuning of the properties CD NPs possess leads to improvement in controlled-targeted drug release and theranostics of most frequently occurring cancers (lung, liver, breast, cervical, and prostate).

Despite the progress nanoscale drug delivery vehicles has achieved, further improvement is necessary to reach the clinical standard of care. CDs are very promising agents, which has proven its potential in playing multiple roles at the nanoscale level. Nonetheless, efforts should be taken to translate the *in vitro* efficiency of the delivery system into clinical standard of care with proper *in vivo* models and strategies. More active targeting approaches must be taken along with the (passive) EPR effect to overcome cancer. Also, exploring the potential of traditional Ayurvedic compositions and plant metabolites for its anticancer activity can help us overcome the current drug-resistant tumors and possibly serve as a more efficient treatment. NPs that combine molecular targeting, imaging capabilities, and therapeutic agents have the potential to become next-generation nanomaterials for effectively detecting and treating cancer. Combined efforts from doctors, molecular biologists, material scientists, medicinal chemists, and biotechnologists shall be important in tackling the cancers in a more personalized way.
